# Generation of DelNS1 Influenza Viruses: a Strategy for Optimizing Live Attenuated Influenza Vaccines

**DOI:** 10.1128/mBio.02180-19

**Published:** 2019-09-17

**Authors:** Pui Wang, Min Zheng, Siu-Ying Lau, Pin Chen, Bobo Wing-Yee Mok, Siwen Liu, Honglian Liu, Xiaofeng Huang, Conor J. Cremin, Wenjun Song, Yixin Chen, Yik-Chun Wong, Haode Huang, Kelvin Kai-Wong To, Zhiwei Chen, Ningshao Xia, Kwok-Yung Yuen, Honglin Chen

**Affiliations:** a State Key Laboratory for Emerging Infectious Diseases, Department of Microbiology, Li Ka Shing Faculty of Medicine, The University of Hong Kong, Hong Kong SAR, People’s Republic of China; b National Institute of Diagnostics and Vaccine Development in Infectious Diseases, School of Public Health, Xiamen University, Xiamen, People’s Republic of China; c State Key Laboratory of Molecular Vaccinology and Molecular Diagnostics, School of Public Health, Xiamen University, Xiamen, People’s Republic of China; d AIDS Institute, Department of Microbiology, Li Ka Shing Faculty of Medicine, The University of Hong Kong, Hong Kong SAR, People’s Republic of China; National Institute of Allergy and Infectious Diseases; National Institute of Allergy and Infectious Diseases; University of Edinburgh

**Keywords:** live attenuated vaccine, NS1, influenza vaccines

## Abstract

Nonstructural protein 1 (NS1) of influenza virus is a key virulence element with multifunctional roles in virus replication and a potent antagonist of host immune response. Deletion of NS1 (DelNS1) would create a safer and more extensively immunogenic live attenuated influenza virus (LAIV) vaccine. However, DelNS1 viruses are very difficult to grow in regular vaccine-producing systems, which has hampered the application of DelNS1 LAIV vaccines in humans. We have developed two master backbones of deleted-NS1 (DelNS1) viral genomes from influenza A or B viruses which contain novel adaptive mutations to support DelNS1-LAIV replication. These DelNS1-LAIVs are highly attenuated in human cells *in vitro* and nonpathogenic in mice but replicate well in vaccine-producing cells. Both influenza A and influenza B DelNS1 LAIVs grow better at 33°C than at 37 to 39°C. Vaccination with DelNS1 LAIV performed once is enough to provide potent protection against lethal challenge with homologous virus and strong long-lasting cross protection against heterosubtypic or antigenically distantly related influenza viruses in mice. Mechanistic investigations revealed that DelNS1-LAIVs induce cross protective neutralizing antibody and CD8^+^ and CD4^+^ T cell immunities. Importantly, it has been shown that DelNS1-LAIV can be used to enhance specific anti-influenza immunity through expression of additional antigens from the deleted-NS1 site. Generation of DelNS1 viruses which are nonpathogenic and able to grow in vaccine-producing systems is an important strategy for making highly immunogenic LAIV vaccines that induce broad cross protective immunity against seasonal and emerging influenza.

## Abstract (precis)

Current seasonal influenza vaccines are suboptimal and low in immunogenicity and do not provide long-lasting immunity and cross protection against influenza virus strains that have antigenically drifted. More-effective influenza vaccines which can induce both humoral immunity and T cell immunity are needed. The NS1 protein of influenza virus is a virulence element and the critical factor for regulation of the host immune response during virus infection. Deletion of the NS1 protein is a strategy to make an optimal LAIV vaccine. However, DelNS1 viruses are very difficult to grow in regular vaccine-producing systems, hampering the application of DelNS1 LAIV vaccines in humans. We have generated a panel of both influenza A and influenza B DelNS1 LAIVs which are able to grow in regular vaccine-producing cells. These DelNS1 LAIV vaccines are completely nonpathogenic, exhibit potent and long-lasting immunity, and can be used to express extra viral antigen to induce cross protective immunity against seasonal and emerging influenza.

## INTRODUCTION

Influenza poses a significant disease burden, causing deaths through annual seasonal epidemics and also during sporadic pandemics, which have occurred four times since 1918. Vaccination is considered the most effective way to alleviate the disease burden and mortality associated with seasonal influenza and is also the method of choice to curb future human pandemics. However, the highly variable nature of hemagglutinin (HA) in circulating strains makes it difficult to predict the evolution of influenza viruses (IVs). An inability to induce long-lasting immunity and the effects of historical influenza virus infections on vaccine-induced immune responses further complicate the current vaccine strategy. Mismatch of seasonal influenza vaccines and circulating strains occurs regularly, resulting in severe influenza seasons (1). It is also recognized that HA-based influenza vaccine technology cannot respond swiftly to emerging pandemic viruses, as evidenced during the 2009 H1N1 pandemic. Therefore, it is imperative to develop influenza vaccines with the ability to induce enduring broadly protective immunity against matched and mismatched seasonal influenza virus strains and that can be produced rapidly in the scenario of an emerging pandemic.

In recent years, new strategies for the development of novel influenza vaccines conferring superior length and breadth of protection have been extensively explored (2–6). One strategy involves developing a vaccine that induces antibodies targeting the more highly conserved stem region of the influenza virus HA protein (7, 8). Attention has also recently been focused on immunity elicited by influenza virus neuraminidase (NA), which is less variable than HA, but the vaccine applications of this approach have not been fully evaluated (9, 10). Besides HA and NA, vaccines based on the conserved ectodomain of matrix protein 2 of influenza virus have been evaluated previously in multiple reports (11, 12). Another strategy in influenza vaccine research focuses on using live attenuated influenza viruses (LAIVs) to mimic natural infection and induce both humoral and cellular immunity to influenza virus. A cold-adapted LAIV (Influenza Mist) has been licensed for use in humans aged 2 to 49 since 2007 (13). While HA-based vaccines aim to induce HA-specific neutralizing or stem-targeting antibodies to block influenza virus, LAIVs elicit multifaceted immune responses through limited virus replication in mucosal epithelial cells (6). Several studies have used various strategies to make different versions of LAIV based on the A/WSN/1933 virus (6, 14–16). Testing of these conceptual vaccines in animal models indicates that they can cross protect against heterologous viral challenges. These LAIVs generally contain mutations or artificial insertions which attenuate their ability to antagonize host cell expression of interferon (IFN) and antiviral activity; the long-term stability of these mutations and the performance of the attenuated phenotypes in humans are not yet certain, and these LAIVs are not practical for mass production, due to the attenuation of virus replication. Nevertheless, LAIVs are consistently able to induce broader cross protective immunity than current inactivated influenza vaccines. It has long been questioned why natural infections with influenza virus fail to induce broad and long-lasting immunity against future encounters with similar viruses. Influenza viruses are known to block or interfere with innate and adaptive host immune responses. Influenza virus nonstructural protein 1 (NS1) is a key virulence element and a strong IFN antagonist, with multifunctional roles in virus replication (see reviews in references 17 and 18). While the NS1 protein is not essential for virus replication, it is critical for evasion of host innate immunity and interference with the induction of adaptive immunity during influenza virus infection (19, 20). A role for NS1 in airborne transmission of avian H1N1 virus between ferrets has also been shown previously (21). Therefore, deletion of NS1 has long been considered a desirable strategy for making safer and more immunogenic influenza vaccines (22). An NS1-truncated vaccine induces strong immune responses and appears to exert better protective efficacy than an inactivated virus vaccine in aged mice (23, 24). LAIVs expressing truncated or mutated NS1 genes in the backbone of H3N2 or H1N1 viruses also cross protect against swine influenza viruses in pigs (25–27), further highlighting the potential of modified NS1 vaccines for use in preventing influenza. Furthermore, two clinical trials using deleted-NS1-trivalent LAIVs containing influenza virus H1N1, H3N2, and B strains demonstrated induction of significant levels of strain-specific and cross-neutralizing antibodies in vaccinated humans (28, 29). However, influenza viruses with truncated or deleted NS1 grow at relatively low levels of efficiency in MDCK cells (30), which has hampered the commercial development of deleted-NS1 (DelNS1) live attenuated influenza virus vaccine strains. Previous studies published by ourselves and others have reported mutations which support the replication of DelNS1 viruses in MDCK cells and/or embryonated chicken eggs (31–33). It seems possible that acquisition of adaptive mutations that restore NS1-regulated M splicing during influenza virus infection would increase virus replication ability in interferon-deficient systems (31), providing a practical way to produce sufficient quantities of DelNS1 viruses for use as live attenuated influenza vaccines. It will also be important to evaluate if additional viral antigens can be expressed from the NS segment in the DelNS1 LAIV system to enhance specific immunity to viral targets.

Extending from our previous work characterizing a DelNS1 virus derived from A/WSN/1933 (31), we have established a panel of DelNS1 versions of influenza A (2009 H1N1, H3N2, and H7N9) and B virus strains based on master donor strains derived from the 2009 H1N1 and HK2011 influenza B virus strains, respectively. We found that different strains of influenza viruses utilize different adaptive strategies to support replication of DelNS1 viruses. These DelNS1 viruses are avirulent in mice, regardless of whether they originated from seasonal, pandemic, or highly pathogenic avian influenza viruses, and are naturally cold-adapted. Importantly, immunization of mice with the H1N1 DelNS1 LAIV vaccine induces long-lasting cross protective immunity against lethal challenge with 2009 pandemic H1N1, H3N2, and avian H5N1 or H7N9 viruses. Broad protection against antigenically distantly related influenza viruses was also observed for DelNS1-H3N2 and DelNS1 B LAIVs. We show that 2009 H1N1-DelNS1 LAIV is a safe and versatile master donor backbone for making vaccine versions of other subtype viruses and for expressing additional antigens to enhance specific immunity against influenza viruses. DelNS1 LAIVs with adaptive mutations enabling growth in high-volume vaccine production systems should be considered an important strategy to be further evaluated in the quest to develop more-effective and broadly protective vaccines for seasonal and emerging pandemic influenza viruses.

## RESULTS

### Generation of 2009 H1N1 DelNS1 virus with cold-adaptive properties.

**FIG 1:**
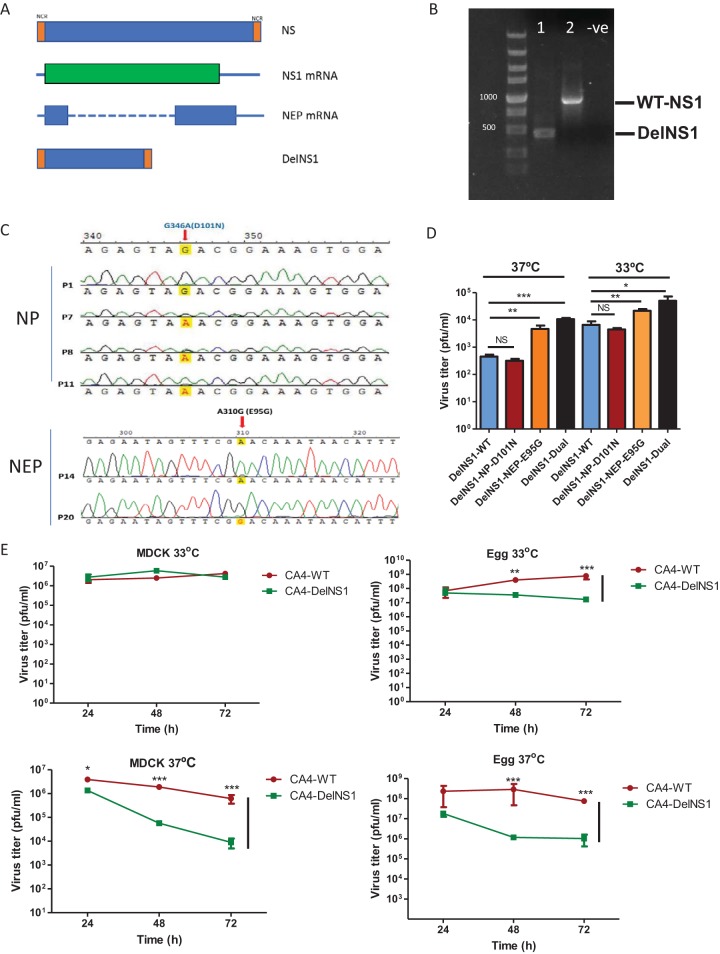
Generation and characterization of CA4-DelNS1 virus. (A) Full-length and NS1 gene-deleted NS segments. NCR, noncoding region. (B) Confirmation of NS1 deletion in rescued CA4-DelNS1 virus by RT-PCR and agarose gel electrophoresis. -ve, negative control. (C) Newly rescued CA4-DelNS1 virus was passaged in MDCK cells 20 times to promote growth adaptation. After the virus titers stabilized, the entire genome of the CA4-DelNS1 virus was analyzed and adaptive mutations were identified. (D) Effect of adaptive mutations on the efficiency of CA4-DelNS1 virus rescue. DelNS1 pHW2000 plasmids with individual or combined adaptive mutations or the original DelNS1 plasmid (DelNS1-WT) were transfected together with plasmids encoding the other 7 viral segments into 293T/MDCK cell mixtures and incubated at 33°C or 37°C. After 72 h, viral supernatants were collected and titrated. (E) Growth of CA4-DelNS1 at 33°C or 37°C was analyzed. MDCK cells or embryonated chicken eggs were infected with CA4-DelNS1 or WT virus at 0.1 MOI or 1,000 PFU, respectively. Viral supernatants or allantoic fluids were collected at the indicated time points and titrated by plaque assay in MDCK cells. Data represent mean values ± standard deviations of results from three independent experiments. Statistical comparisons between means were performed by Student's *t* test. ***, *P* < 0.001; **, *P* < 0.01; *, *P* < 0.05; NS, not significant.

We previously identified a unique mutation (A14U) in the M segment which enhances replication of A/WSN/1933 and A/PR8/1934 H1N1 DelNS1 viruses in interferon-deficient cells (31). Since the WSN and PR8 strains are no longer circulating among humans, we elected to use a relatively contemporary H1N1 strain to serve as the backbone for construction of DelNS1 vaccine strains. The 2009 H1N1 virus causes only mild disease in humans and has become one of the two prevalent seasonal influenza A viruses. We consider this to be an ideal virus from which to make a backbone for influenza A DelNS1 LAIV. Using technology similar to that previously described, a DelNS1 version of the A/CA/04/2009 (CA4) virus where the NS1 is removed but the NEP is preserved was constructed and deletion of the NS1 coding region from the NS segment verified (Fig. 1A and B). The NS1-deleted version of the A/CA/04/2009 (H1N1) virus was rescued using a reverse genetics procedure and passaged in MDCK cells 10 times at 37°C and then a further 10 times at 30°C as previously described (31). The resultant passage-adapted virus was designated CA4-DelNS1, and that name was used in subsequent experiments and throughout this report. Two adaptive mutations, located in the NP (D101N) and NEP (E95G) genes, were identified in the resulting growth-adapted CA4-DelNS1 virus (Fig. 1C). The requirement for both adaptive mutations for DelNS1 virus replication was investigated by constructing DelNS1 gene segments containing the mutations alone or in combination and examining the efficiency of reverse genetics virus rescue from plasmid transfection (Fig. 1D). The paired adaptive mutations promoted more efficient rescue than either mutation alone, and, interestingly, the efficiency of rescue at 33°C was higher than at 37°C (Fig. 1D). Growth kinetics analysis showed that the stably growth-adapted CA4-DelNS1 virus had gained the ability to replicate comparably to the wild-type (WT) virus following more than 20 passages in MDCK cells (Fig. 1E). We then performed growth kinetics analysis of CA4-DelNS1 and the WT parent virus in MDCK cells and embryonated chicken eggs and found that DelNS1 virus replicated similarly to WT virus in MDCK cells and slightly less well than WT virus in eggs at 33°C, whereas DelNS1 replication efficiency was lower than that of WT virus in both culture systems at 37°C (Fig. 1E). The preference of CA4-DelNS1 viruses for replication at the lower temperature of 33°C, rather than 37°C, was entirely spontaneous and is a desirable feature in a live attenuated influenza virus vaccine. To further verify the contribution of the mutations in NP and NEP to growth and temperature adaptation, the growth kinetics of CA4-DelNS1 viruses carrying single or double mutations were examined, revealing that both mutations enhanced CA4-DelNS1 replication and facilitated cold adaptation in MDCK cells and embryonated chicken eggs, with the effect of NEP E95G being more apparent with respect to supporting CA4-DelNS1 virus growth (see Fig. S1 in the supplemental material). These results show that the presence of adaptive mutations in the NP (D101N) and NEP (E95G) genes supported the replication of CA4-DelNS1 virus to levels nearly equal to those measured for the WT virus in MDCK cells and chicken eggs.

10.1128/mBio.02180-19.1 FIG S1 Effect of the NP-G346A(D101N) and NEP-A310G(E95G) adaptive mutations on the growth of CA4-DelNS1 virus in MDCK cells and embryonated chicken eggs. Download FIG S1, PDF file, 0.1 MB. Copyright © 2019 Wang et al. 2019 Wang et al. This content is distributed under the terms of the Creative Commons Attribution 4.0 International license.

### Avirulence of DelNS1 viruses *in vitro* and *in vivo*.

**FIG 2:**
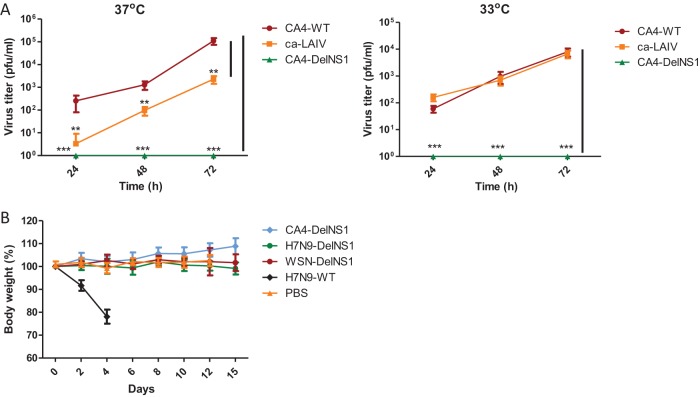
Avirulence of CA4-DelNS1 virus. (A) A549 cells were infected with CA4-WT virus, with ca-LAIV, or with CA4-DelNS1 virus at 0.01 MOI and cultured at 33°C or 37°C. ca-LAIV is a cold-adapted 2009 H1N1 virus that was isolated from the licensed LAIV vaccine (17). Viral supernatants were collected at different time points and titrated by plaque assay in MDCK cells. Data represent mean values ± standard deviations of results from three independent experiments. Statistical comparisons between means were performed by Student's *t* test. ***, *P* < 0.001; **, *P* < 0.01. (B) DelNS1 virus infection in mice. Groups of six 6-week-old mice were inoculated with 10^6^ PFU of CA4-DelNS1, H7N9-DelNS1, WSN-DelNS1, or H7N9 WT virus. PBS was used as an inoculation control. Body weight and survival were observed for 14 days. Data represent mean body weight values ± standard deviations of results from 6 mice.

It is essential for any vaccine to be nonpathogenic to humans. We examined replication of the CA4-DelNS1 virus generated in this study, comparing it with that of CA4-WT virus and the licensed cold-adapted H1N1 virus (ca-LAIV), which has different internal genes but has HA and NA similar to those of the CA4 virus. While CA4-WT and ca-LAIV replicated at both 33°C and 37°C in A549 cells, replication of CA4-DelNS1 was undetectable (Fig. 2A), confirming the attenuation of CA4-DelNS1 replication in interferon-competent cells. We then examined the panel of DelNS1 viruses made in our study, inoculating mice with 10^6^ PFU of CA4-DelNS1, H7N9-DelNS1, or WSN-DelNS1 LAIV. Whereas mice inoculated with wild-type H7N9 (A/Zhejiang/DTID-ZJU01/2013) died within 1 week, none of the mice inoculated with any of the DelNS1 viruses died, and no symptoms of body weight loss or disease were observed in these mice (Fig. 2B). These results demonstrate that deletion of NS1 significantly attenuates the pathogenic properties of both seasonal and highly pathogenic avian influenza viruses.

### CA4-DelNS1 H1N1 virus provides protection against homologous and heterologous virus challenge.

**FIG 3:**
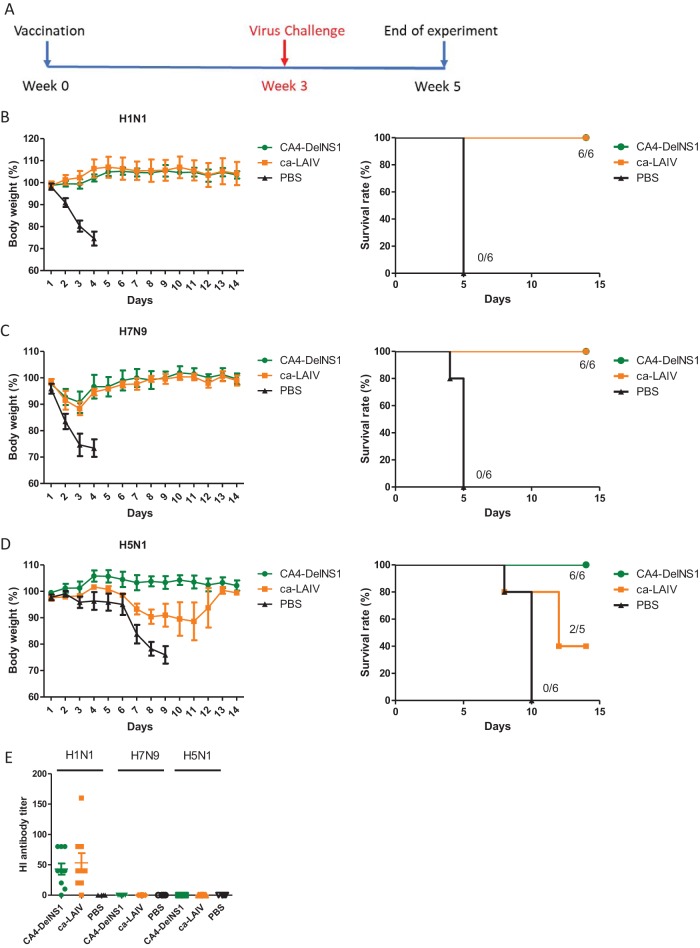
CA4-DelNS1 LAIV vaccination protects mice from both homologous and heterologous virus challenge. (A) Timelines for vaccination and virus challenge. Mice were intranasally vaccinated once with CA4-DelNS1 virus (2 × 10^6^ PFU), ca-LAIV (2 × 10^6^ PFU), or PBS and then challenged with virus after 3 weeks. (B to D) Vaccinated mice were challenged with 10 MLD_50_ CA4 (H1N1) mouse-adapted virus (B), 10 MLD_50_ H7N9 virus (C), or 100 MLD_50_ H5N1 virus (D). Body weights and survival were observed for 14 days. Body weight data represent mean values ± standard deviations of results from 6 mice. (E) HI analysis of sera from mice vaccinated with CA4-DelNS1, ca-LAIV, or PBS, collected prior to virus challenge.

**FIG 4:**
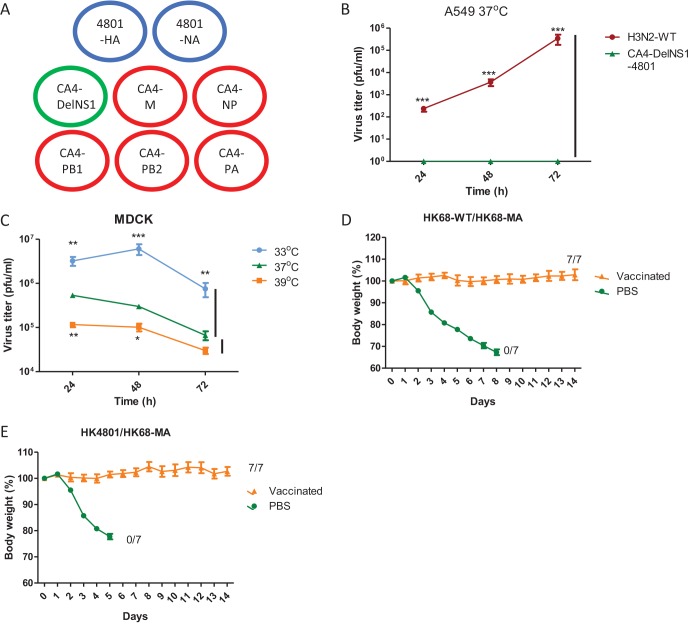
CA4-DelNS1 as a master backbone for construction of DelNS1 H3N2 LAIV. (A) Generation of CA4-DelNS1-HK4801 (H3N2) by plasmid transfection. CA4-DelNS1-HK4801 (H3N2) virus was rescued with HA and NA plasmids derived from the A/Hong Kong/4801/2014 (H3N2) strain and 6 internal gene plasmids from the CA4-DelNS1 virus containing adaptive mutations, as described for Fig. 1. (B) CA4-DelNS1-HK4801 cannot replicate in IFN-competent A549 cells. (C) CA4-DelNS1-HK4801 replication efficiency varied with temperature in MDCK cells. (D) CA4-DelNS1-HK68 (H3N2) protects mice from challenge with homologous HK68-MA virus. Mice were vaccinated with CA4-DelNS1-HK68 (HK68-WT) virus (2 × 10^6^ PFU) or PBS and after 3 weeks were challenged with HK68-MA (mouse-adapted) virus. (E) CA4-DelNS1-HK4801 protects mice from challenge with heterologous HK68-MA virus. Mice were vaccinated with CA4-DelNS1-HK4801 (H3N2) virus (3 × 10^7^ PFU) or PBS and were challenged later with HK68-MA virus. Body weight and survival were observed for 14 days. Body weight data represent mean values ± standard deviations of results from 6/7 mice. Statistical comparisons between means were performed by Student's *t* test. ***, *P* < 0.001; **, *P* < 0.01; *, *P* < 0.05.

To assess the protective ability of DelNS1 virus in influenza virus infection, we immunized mice once with CA4-DelNS1 virus, through the nasal route. At 3 weeks after immunization, mice were challenged with mouse-adapted H1N1 virus or with H7N9 or H5N1 viruses (Fig. 3A). To understand the effectiveness of DelNS1 virus in inducing host immunity, the ca-LAIV H1N1 virus was included as a comparison. Animals were challenged with lethal doses of viruses, as indicated, and followed for 2 weeks after infection. Immunization with either CA4-DelNS1 virus or ca-LAIV fully protected against lethal challenge with mouse-adapted H1N1 virus (Fig. 3B). No virus was detected in the lung tissues of mice from vaccinated groups at day 3 postinfection (Fig. S3A). Interestingly, immunization with CA4-DelNS1 virus and ca-LAIV also protected against a H7N9 virus challenge with 10 50% murine lethal doses (MLD_50_), with mice displaying only slight body weight loss during the first 3 days of infection, followed by full recovery (Fig. 3C). Consistent with this body weight loss, H7N9 virus was still actively replicating in the lungs of vaccinated mice at day 3 postchallenge (Fig. 3B). Highly pathogenic avian influenza H5N1 virus infection is fatal in approximately 60% of human cases. It is noteworthy that immunization with CA4-DelNS1 virus provided full protection to H5N1 virus-challenged mice, with no deaths or body weight loss observed in any of these animals (Fig. 3D). Cross protection was also observed with the ca-LAIV vaccine, but it conferred only partial protection, with 3/5 mice dying and the surviving mice (2/5) experiencing significant body weight loss (Fig. 3D), further demonstrating that CA4-DelNS1 LAIV provides better cross protective activity than ca-LAIV, which contains an intact NS1 gene. However, virus titers in the lungs of challenged mice were not significantly different (Fig. S3A to C). Cross protective immunity against antigenically distinct influenza A strains was also induced by CA4-DelNS1 (H1N1) and CA4-DelNS1-HK4801, with the latter bearing HA and NA from the H3N2 A/HK/4801/2014 virus on the CA4-DelNS1 backbone, in a separate experiment where LAIV-vaccinated mice were challenged with mouse-adapted A/HK/1/68 (H3N2) virus (Fig. 4A; see also Fig. S3D and E). To investigate if cross-reactive antibodies are induced by immunization, sera from immunized mice were collected prior to virus challenge and analyzed by hemagglutinin inhibition (HI) assay. Both CA4-DelNS1 and ca-LAIV induced a fair level of antibodies against the H1N1 virus, but antibodies specific for either H7N9 or H5N1 were undetectable (Fig. 3E), suggesting that the observed cross protective immunity might be facilitated by an immunological mediator other than HA-specific antibodies. To examine if the cross protective immunity mediated by DelNS1 LAIV is long-lasting, mice were immunized 10 weeks before challenge. Cross protection was observed following challenge with H1N1, H7N9, and H5N1 viruses, and the level of protection shown by CA4-DelNS1 virus against H5N1 virus was superior to that shown by ca-LAIV (Fig. S2A to D). To test whether DelNS1 LAIV might produce defective interfering (DI) virus as cross protective antiviral agents (34), we compared DI viral genomes from DelNS1 LAIV and the H1N1 vaccine strain isolated from Flumist LAIV season vaccine (2014 to 2015) passaged in eggs. While statistically significant levels of DI genomes from PB2 and PA were detected from a commercial LAIV H1N1 strain, no significant DI genomes were detected from either influenza A or influenza B DelNS1 LAIV (Fig. S2E). These results show that DelNS1 live attenuated influenza virus induces sustained and highly protective immunity against lethal challenge with both homologous and heterologous subtype viruses and that deletion of NS1 from CA4 created a live attenuated influenza vaccine that seems to be more cross protective than the ca-LAIV which contains wild-type NS1.

10.1128/mBio.02180-19.2 FIG S2 CA4-DelNS1 LAIV vaccination cross protects mice from both homologous and heterologous virus challenge. Download FIG S2, PDF file, 0.2 MB. Copyright © 2019 Wang et al. 2019 Wang et al. This content is distributed under the terms of the Creative Commons Attribution 4.0 International license.

10.1128/mBio.02180-19.3 FIG S3 Viral titers in lungs of mice infected with H1N1, H7N9, H5N1, and H3N2 (HK68) viruses. Download FIG S3, PDF file, 0.1 MB. Copyright © 2019 Wang et al. 2019 Wang et al. This content is distributed under the terms of the Creative Commons Attribution 4.0 International license.

### DelNS1 H3N2 live attenuated virus confers broad protection against antigenically drifted virus.

To assess the suitability of the CA4-DelNS1 virus for use as a master donor backbone, we constructed a DelNS1-H3N2 LAIV using the CA4-DelNS1 backbone and HA and NA derived from the A/HK/4801/2014 H3N2 virus. The NS1-deleted version of the A/HK/4801/2014 (H3N2) virus was rescued with the six internal genome segments of the CA4-DelNS1 (H1N1) virus, which carry the adaptive mutations for virus growth and temperature restriction (Fig. 4A) (here designated CA4-DelNS1-HK4801 in subsequent descriptions of experiments). As anticipated, CA4-DelNS1-HK4801 (H3N2) virus is unable to replicate in A549 cells (Fig. 4B). Analysis of growth properties in MDCK cells showed that CA4-DelNS1-HK4801 (H3N2) LAIV also exhibits a preference for replication at 33°C, with growth being restricted at 37°C and 39°C (Fig. 4C). This demonstrates that CA4-DelNS1 can be used as a donor master strain for the construction of other HA and NA subtype DelNS1 LAIVs. There are very few mouse-adapted H3N2 viruses available for use in challenge experiments. To assess the protective activity of the CA4-DelNS1-HK4801 (H3N2) LAIV, we challenged mice with a mouse-adapted virus derived from a 1968 H3N2 isolate and also created a DelNS1 version of the original non-mouse-adapted isolate (CA4-DelNS1-HK68) (35, 36) for use in subsequent comparisons. Mice were immunized once with CA4-DelNS1-HK4801 (H3N2) or CA4-DelNS1-HK68 (H3N2) live attenuated virus and then challenged with a lethal dose of the mouse-adapted H3N2 (HK68-MA) strain 3 weeks later. As expected, mice immunized with CA4-DelNS1-HK68 were fully protected from HK68-MA virus challenge (Fig. 4D). Notably, mice immunized with the CA4-DelNS1-HK4801 (H3N2) LAIV, which carries HA and NA whose origins are separated by nearly 50 years from those of the challenging HK68-MA virus strain, were completely protected, with no apparent body weight loss or other disease symptoms being observed (Fig. 4E) and with significantly lower virus titers in lungs at day 3 postinfection (Fig. S4A). HI analysis showed that antibodies reactive to the HK4801 strain were induced prior to virus challenge (Fig. S4B). However, these antibodies were not reactive to the HK68 strain or to a more recent H3N2 vaccine strain, A/Brisbane/10/2007 (Bris10), similarly to the results seen with CA4-DelNS1 H1N1 LAIV (Fig. S2E). HI tests revealed a low but detectable level of antibodies reactive to the HK68 strain present in sera collected from CA4-DelNS1-HK4801 LAIV-protected mice 2 weeks after HK68-MA virus challenge. Neutralization tests confirmed the presence of neutralizing antibodies corresponding to both HK4801 and HK68-MA in CA4-DelNS1-HK4801 LAIV-immunized mice prior to challenge and at 2 weeks after challenge (Fig. S4C). Note that levels of both hemagglutinin inhibition and neutralization antibodies recognizing HK4801 were higher after HK68-MA virus challenge, suggesting that HK68-MA virus infection boosts the levels of antibodies initially induced by HK4801 immunization. Given that HK68-MA and HK4801 are antigenically distinct, it is possible that immunization with CA4-DelNS1-HK4801 induces a broad antibody repertoire reactive to conserved epitopes present in both strains and that these antibody repertoires are boosted following subsequent challenge with HK68-MA virus. However, the use of HK68-MA (mouse-adapted) H3N2 virus to study cross protection against antigenic drift strain has limitations, since the vaccinated strain (HK4801) evolved from it. Mouse-adapted H3N2 viruses of more recently isolated H3N2 virus strains are needed to address this issue in future. Taken together, these results show that CA4-DelNS1 virus can be used as a donor backbone for making DelNS1 viruses of other subtypes which are temperature sensitive but able to replicate efficiently in MDCK cells and embryonated chicken eggs. Importantly, recombinant CA4-DelNS1-HK4801 (H3N2) LAIV is highly protective against challenge with antigenically distantly related viral strains. This finding is potentially important, as it suggests that DelNS1 LAIVs can elicit immunity that is sufficiently broad to overcome influenza vaccine mismatches caused by antigenic drift of seasonal influenza viruses.

10.1128/mBio.02180-19.4 FIG S4 Viral titers in lungs of mice infected with HK68-MA virus and HI and neutralizing antibodies in sera before and after virus challenge. Download FIG S4, PDF file, 0.1 MB. Copyright © 2019 Wang et al. 2019 Wang et al. This content is distributed under the terms of the Creative Commons Attribution 4.0 International license.

### DelNS1 influenza B virus cross protects against different genetic lineages.

**FIG 5:**
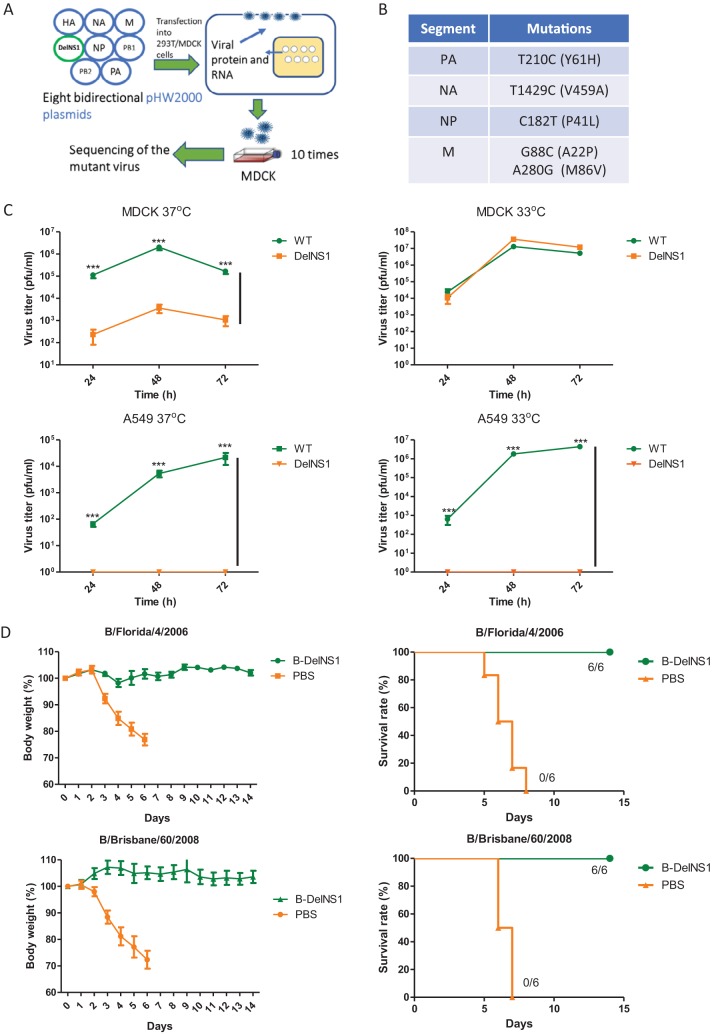
Generation and characterization of DelNS1 B LAIV. (A) Generation of DelNS1-B8038 influenza B virus by reverse genetics. pHW2000 plasmids containing an NS1-deleted (DelNS1) segment and the seven remaining genome segments derived from B/Hong Kong/8038/2011 (Victoria) virus were transfected into a 293T/MDCK cell mixture. Rescued virus was passaged in MDCK cells until the virus titer stabilized. (B) Adaptive mutations in growth-adapted DelNS1-B8038 influenza B virus. Mutations in PA (T210C), NA (T1429C), NP (C182T), and M (G88C plus A280G) were identified. (C) Growth of DelNS1-B8038 influenza B virus in cell culture. MDCK or A549 cells were infected with either WT or DelNS1-B8038 virus at 0.01 MOI and incubated at 33°C or 37°C for 72 h. Virus supernatants were collected and titrated by plaque assay in MDCK cells. Data represent mean values ± standard deviations of results from three independent experiments. Statistical comparisons between means were performed by Student's *t* test. ***, *P* < 0.001. (D) DelNS1-B8038 (B-DelNS1) LAIV vaccination protected mice from challenge with both Victoria and Yamagata lineage mouse-adapted viruses. Mice were vaccinated with DelNS1-B8038 (2 × 10^6^ PFU) or PBS. After 3 weeks, vaccinated mice were challenged with mouse-adapted B/Brisbane/60/2008 (Victoria) or B/Florida/4/2006 (Yamagata) influenza B virus at 10 MLD_50_. Body weight and survival were observed for 14 days. Body weight data represent mean values ± standard deviations of results from 6 mice.

To further evaluate the potential of DelNS1 LAIVs for use in influenza vaccine applications, we generated a DelNS1 influenza B virus using a master donor backbone from a Victoria lineage influenza B virus, B/Hong Kong/8038/2011. The NS1 coding region was deleted from the NS segment of B/Hong Kong/8038/2011, and a NS1-deleted version of influenza B virus was rescued using a reverse genetics protocol (Fig. 5A) as described above. A NS1-deleted version of B/Hong Kong/8038/2011 influenza B virus adapted to replicate in MDCK cells and embryonated chicken eggs was obtained using a similar approach, and the resultant virus was designated DelNS1-B8038. Sequence analysis identified five unique mutations that were present in the PA (T210C/Y61H), NA (T1429C/V459A), NP (C182T/P41L), and M (G88C/A22P and A280G/M86V) genes of the DelNS1-B8038 LAIV genome (Fig. 5B). The effect of these mutations on the growth of DelNS1-B8038 was verified by testing mutations individually or in combination in a transfection assay (Fig. S5A). Analysis of growth properties found that DelNS1-B8038 influenza B virus replicates at 33°C in MDCK cells but exhibits reduced replication efficiency at 37°C (Fig. 5C), similarly to CA4-DelNS1 influenza A viruses. DelNS1-B8038 LAIV cannot replicate in interferon-competent A549 cells (Fig. 5C). There are two genetically distinct lineages of influenza B viruses, namely, Victoria and Yamagata, cocirculating in humans. To assess if DelNS1-B8038, a Victoria lineage influenza B virus, provides protection from infection with both lineages of influenza B virus, mice were immunized once through the nasal route and were then (after 3 weeks) challenged with mouse-adapted influenza B virus of either the Yamagata (B/Florida/4/2006) or Victoria (B/Brisbane/60/2008) lineage (37). Mice mock vaccinated with phosphate-buffered saline (PBS) died after 6 to 8 days, whereas all DelNS1-B8038 LAIV-immunized mice survived, with only minor body weight loss observed in the B/Florida/4/2008 (Yamagata) challenge group and no apparent disease symptoms or body weight loss observed in the B/Brisbane/60/2008 challenge group (Fig. 5D). Virus titers in lungs were significantly lower in DelNS1-B8038 LAIV-vaccinated mice than in the mice in the control group at day 3 postinfection (Fig. S5B). These results show that the DelNS1 B LAIV exhibits favorable cold adaptation and temperature restriction properties and is able to induce cross protective activity against antigenically distinct influenza B virus strains.

10.1128/mBio.02180-19.5 FIG S5 Viral titers in lungs of mice infected with mouse-adapted influenza B viruses. Download FIG S5, PDF file, 0.1 MB. Copyright © 2019 Wang et al. 2019 Wang et al. This content is distributed under the terms of the Creative Commons Attribution 4.0 International license.

### DelNS1 LAIV induces cross protective T cell immunity.

**FIG 6:**
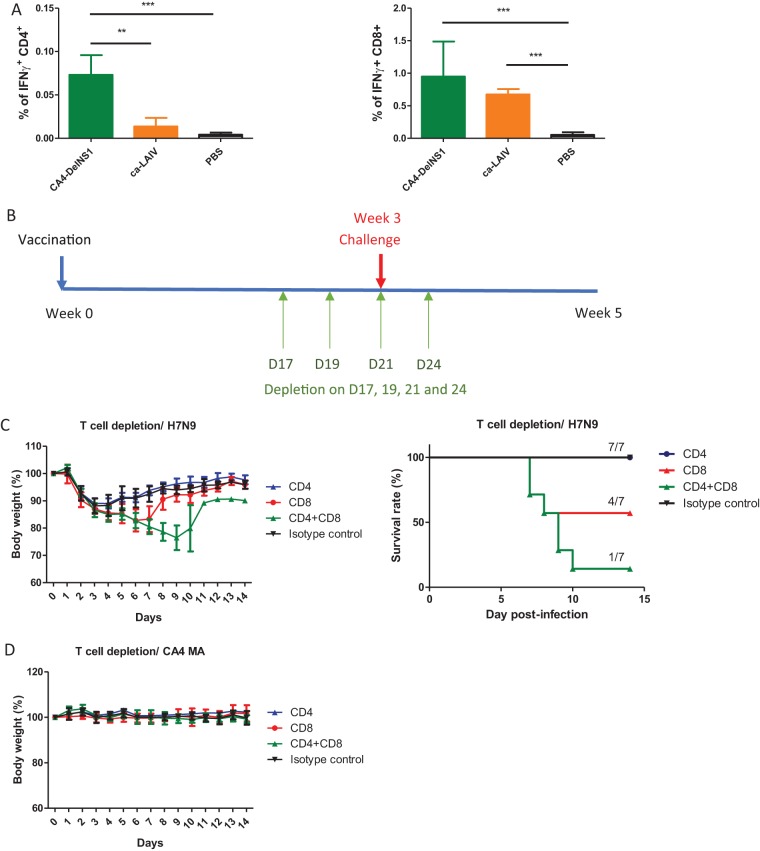
DelNS1 LAIV induces cross protective T cell responses against influenza viruses. (A) Induction of antigen-specific CD8^+^ and CD4^+^ T cells by CA4-DelNS1 LAIV was estimated by intracellular cytokine staining (ICS) of *ex vivo* peptide-stimulated splenocytes, followed by flow cytometric analysis. (B) Schedule of T cell depletion and virus challenge. Vaccinated mice were injected intraperitoneally (i.p.) with 100 μg of anti-CD8α or anti-CD4 or with both anti-CD8α and anti-CD4 or with isotype control (IgG2b) antibodies on day 17 (D17), D19, and D21 after immunization and day 3 after virus challenge. (C) Mice were challenged with H7N9 virus (10 MLD_50_) on day 21 after vaccination and monitored for 14 days. (D) Mice were challenged with mouse-adapted CA4 (H1N1) virus (10 MLD_50_) and monitored for 14 days. Body weight data represent mean values ± standard deviations of results from 7 mice. Statistical comparisons between means were performed by Student's *t* test. ***, *P* < 0.001; **, *P* < 0.01.

The results described above suggested that the cross protective immunity induced by CA4-DelNS1 (H1N1) may be attributable to non-HA-specific antibodies or cellular immunity. To verify the role of T cell immunity following DelNS1 LAIV immunization, we first evaluated CD4^+^ and CD8^+^ responses in CA4-DelNS1 virus-immunized and ca-LAIV-immunized mice. CD4^+^ and CD8^+^ T cells specific for conserved influenza virus (NP) epitopes were analyzed using intracellular cytokine staining (ICS) of splenocytes from immunized mice. Both CA4-DelNS1 and ca-LAIV H1N1 viruses were found to have induced NP-specific CD4^+^ and CD8^+^ T responses 3 weeks after a single intranasal dose (Fig. 6A; see also Fig. S6A and B). It appears that CA4-DelNS1 induces significantly higher levels of CD4^+^ T cells than ca-LAIV. However, it remains to be determined if this difference may be caused by the single amino acid difference between the CD4 epitopes in the NP of ca-LAIV and CA4-DelNS1 virus. To determine if specific CD4^+^ and CD8^+^ T cells are critical to the cross protection provided by CA4-DelNS1 (H1N1) LAIV, immunized mice were treated with anti-CD4 or anti-CD8 antibodies, singly or in combination, to deplete these T cell subsets and were then challenged with a lethal dose of H7N9 virus (Fig. 6B). Depletion of T cells was confirmed 1 day before virus challenge to ensure that more than 98% depletion was achieved compared with the isotype control (Fig. S6C). LAIV-immunized mice with subsequent CD4^+^ and/or CD8^+^ T cell depletion were then challenged with H7N9 virus (10 MLD_50_) and survival and body weight change monitored for 14 days. Combined depletion of CD4^+^ and CD8^+^ T cells severely compromised CD4-DelNS1-immunized mice, with only 1/7 of mice surviving and with these mice showing significant body weight loss and high virus titers in lungs (Fig. 6C; see also Fig. S6D). While both CD8^+^ and CD4^+^ T cells are required for optimal cross protection, CD8^+^ may be the more critical of the two for mediating cross protective cell immunity against influenza virus infection. However, when CA4-DelNS1 LAIV-immunized mice were depleted of CD4^+^ and/or CD8^+^ T cells and then challenged with a lethal dose of H1N1 virus, all mice remained fully protected and showed no sign of disease or weight loss (Fig. 6D). The data clearly demonstrate that DelNS1 LAIV induced both antibody and T cell immunities and that cross protective immunity was mainly mediated by influenza virus-specific T cells.

10.1128/mBio.02180-19.6 FIG S6 Depletion of CD4^+^ and CD8^+^ T cells from CA4-DelNS1 LAIV-immunized mice and virus titers in lungs of mice challenged with H7N9 virus. Download FIG S6, PDF file, 0.4 MB. Copyright © 2019 Wang et al. 2019 Wang et al. This content is distributed under the terms of the Creative Commons Attribution 4.0 International license.

### Delivery of additional viral antigens using the DelNS1 LAIV system.

**FIG 7:**
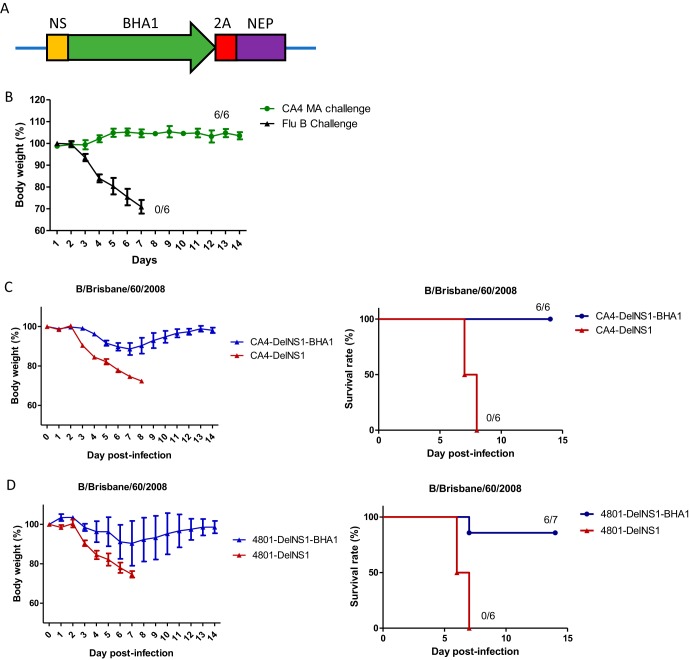
DelNS1 LAIV expression of B-HA1 antigen protected against lethal challenge with both influenza A and B viruses in mice. (A) Illustration of cloning of B-HAI region into the CA4-DelNS1 NS segment. (B) Mice were vaccinated once with CA4-DelNS1 and then challenged 3 weeks later with mouse-adapted H1N1 influenza A virus (A/California/04/2009, 10 MLD_50_) or influenza B virus (B/Brisbane/60/2008, 10 MLD_50_) and monitored for 14 days. (C) Mice were vaccinated twice, 1 month apart, with 10^6^ PFU of either CA4-DelNS1 virus or CA4-DelNS1-BHA1, which contains the HA1 gene of influenza B virus (B/Brisbane/60/2008) at the site in the NS segment from which NS1 was deleted. Mice were challenged with mouse-adapted B/Brisbane/60/2008 (10 MLD_50_) 1 month after the second vaccination and monitored for 2 weeks. (D) Mice were vaccinated once with 1 × 10^7^ PFU of either CA4-DelNS1-HK4801 (H3N2) virus or CA4-DelNS1-HK4801-BHA1 LAIV. Vaccinated mice were challenged with mouse-adapted B/Brisbane/60/2008 (10 MLD_50_) 1 month after the second dose of vaccination. Mice were monitored for body weight change and survival for 2 weeks. Body weight data represent mean values ± standard deviations of results from 6 (B and C) or 7 (D) mice.

While CA4-DelNS1 (H1N1) immunization induced cross protective immunity against challenge with H7N9, H5N1, and HK68 (H3N2) influenza A viruses (Fig. 3; see also Fig. S2 and S3), no protection was observed when mice were challenged with influenza B virus (Fig. 7B). This indicates that the cross protective immunity induced by CA4-DelNS1 (H1N1) LAIV is influenza A specific and does not extend to other types of influenza virus or to other viruses. Since NS1 is deleted from the viral genome in DelNS1 LAIV, there is an opportunity to express a new or additional antigen in its place. We tested this possibility by expressing the influenza B HA1 domain in the NS1 site of the CA4-DelNS1 LAIV genome to generate a chimeric virus, CA4-DelNS1-BHA1, expressing BHA1 from the CA4-DelNS1 (H1N1) backbone (Fig. 7A). Expression of influenza B virus HA1 was confirmed by Western blot analysis, and the genome of the chimeric CA4-DelNS1-BHA1 virus found to be stable during continuous passage in MDCK cells (Fig. S7A and B). Notably, full protection was observed when mice immunized with two doses of CA4-DelNS1-BHA1 virus were challenged with a lethal dose of mouse-adapted B/Brisbane/60/2008 virus, while all mice vaccinated with CA4-DelNS1 LAIV without B-HA1 died (Fig. 7C). Using an H3N2 version, CA4-DelNS1-HK4801-BHA1 LAIV, 6 of 7 mice in the group immunized with vaccine expressing the influenza B HA1 antigen survived, while all mice immunized with one dose of vaccine without additional BHA1 died (Fig. 7D). These data highlight a novel strategy to improve the current influenza vaccine regime by using DelNS1 LAIV to express additional viral antigens and thereby broaden and enhance specific immunity to influenza viruses.

10.1128/mBio.02180-19.7 FIG S7 Construction of CA4-DelNS1 (H1N1) LAIV expressing influenza B virus HA1. Download FIG S7, PDF file, 0.2 MB. Copyright © 2019 Wang et al. 2019 Wang et al. This content is distributed under the terms of the Creative Commons Attribution 4.0 International license.

## DISCUSSION

Currently available inactivated influenza vaccines are suboptimal, particularly in the groups that are most highly at risk, and do not induce sufficiently broad cross protective activity to protect against strains that have antigenically drifted (38, 39). Alternative strategies for the development of more highly effective and cross protective and long-lasting influenza vaccines must be pursued. Influenza viruses in which the NS1 protein is deleted are avirulent and are postulated to be more immunogenic, due to the absence of the immune response suppression usually mediated by the NS1 protein. With this assumption, we established a panel of DelNS1 live attenuated influenza viruses (LAIVs) bearing adaptive mutations that support their replication in MDCK cells and embryonated chicken eggs, enabling large-scale production without helper virus assistance. Master donor backbone viruses were established for both influenza A virus and influenza B virus and were verified in *in vitro* and *in vivo* systems. The currently available LAIV vaccine utilizes cold adaptation and temperature restriction to ensure attenuation in humans. We found that DelNS1 LAIVs are nonpathogenic and have a spontaneous preference for replication at the lower temperature of 33°C. Given the rapid evolution of influenza viruses and the uncertainty regarding the emergence of novel strains, future influenza vaccines should ideally provide cross protection against antigenically drifted viral strains and novel subtypes of zoonotic influenza viruses. Our data strongly support the use of a strategy involving removal of the NS1 protein, a viral antagonist of the host immune response, to make DelNS1 LAIV vaccines which induce more-effective and broader immunity for protection against seasonal and pandemic influenza virus strains. We have shown DelNS1 H1N1, H3N2, and B LAIVs to possess marked cross protective activities in terms of cross subtype and/or antigenically drifted variants in mouse challenge experiments. Importantly, we have also tested a strategy to enhance specific immunity through expression of additional viral antigens in the DelNS1 LAIV system. These findings clearly demonstrate that DelNS1 LAIVs with adaptive mutations that support replication in MDCK cells and embryonated chicken eggs and the capacity to express extra viral antigens will make better influenza vaccines, addressing some critical pitfalls of existing products.

One of the advantages of LAIV vaccines over inactivated split or subunit influenza virus vaccines is the ability to induce both humoral and cellular immunity. The currently available commercial LAIV (Influenza Mist) is based on cold adaptation/temperature sensitivity/attenuation (ca/ts/att) phenotypic features which allow the vaccine virus to replicate at the lower temperatures experienced in the upper respiratory tract but which restrict or attenuate replication at the higher temperature within the lungs (40). LAIVs have been shown to induce a significant amount of mucosal IgA but modest levels of serum IgG (41, 42). Blocking virus transmission is one of the key criteria for seasonal and pandemic influenza vaccines. The superior ability of LAIVs to induce potent mucosal immunity could be important for limiting virus transmission, especially when used in response to a pandemic or a severe epidemic. However, this cold-adapted LAIV vaccine replicates so efficiently in the upper respiratory tract of young children who are seronegative or who have not experienced previous infection with influenza viruses, being shed at a comparatively high titer for a long period of time (41), that it is not recommended for use in children below 2 years old, who are commonly naive with respect to influenza virus infection. On the other hand, preexisting immunity from previous exposure to similar viruses may limit the replication of the current LAIV vaccine in adults. Therefore, an improved LAIV with a lower level of associated virus shedding that more potently induces a full spectrum of protective immunity in the elderly is necessary. Other strategies used to construct different forms of LAIV include acquisition of interferon-sensitive mutations and use of premature termination codons to attenuate or completely block influenza virus replication in normal cells (6, 14–16). Those studies were performed mainly on the basis of the genetically well-characterized A/WSN/1933 or A/PR8/1934 H1N1 strains, and it is not clear if such strategies can be applied in other subtypes of influenza A or influenza B viruses, given that strain-specific adaptive mechanisms are relatively common in influenza viruses. There is also concern regarding the stability of attenuation and the risk of reversion resulting in the virulence of original strains being regained during infection with LAIV vaccines. Live attenuated influenza vaccines with modified NS1 or lacking the entire NS1 coding region have been extensively explored and evaluated in two previous human trials (22, 28, 29). DelNS1 LAIV is made by permanently removing the *NS1* coding region from the viral genome, making the resultant vaccine virus fully attenuated and unable to cause disease in the host. The NS1 protein has important roles in virus replication, and the introduction of a novel NS segment would make the constellation of viral genes unfit for replication. As such, the risk of DelNS1 LAIV reassortment with a recently emerged novel strain to regain the NS1 in the situation of a pandemic is greatly minimized. However, the lack of an effective production system has limited the further development of modified and NS1-deleted vaccines. The panel of DelNS1 LAIVs described here contained adaptive mutations facilitating efficient replication in vaccine-producing MDCK cells or in embryonated eggs. These DelNS1 LAIVs are not expected to release significant levels of virus particles from normal epithelial cells in the upper respiratory tract, as suggested by the attenuation seen in *in vitro* cultures of A549 cells (Fig. 2A). This property will make the DelNS1 virus safer than the current LAIV vaccine, preventing excess shedding of virus following immunization of immunologically naive or seronegative young children or of individuals who had not previously been infected with influenza virus. The safety features inherent with the removal of a key virulence element, together with the advantage of inducing more-potent immunity in the absence of NS1 interference, make DelNS1 LAIV a much-improved version of the LAIV vaccine for use against both seasonal and pandemic influenza virus strains.

Influenza virus is unique in its ability to infect multiple host species and to sustain circulation in humans despite limited genome coding capacity, which may be partly attributable to the plasticity of the hemagglutinin and NS1 proteins (43, 44). Influenza viruses utilize the multiple functions of the NS1 protein to modulate host innate and adaptive immunity, allowing reinfection of humans and mediation of host adaptation (17, 18, 45). Deletion of the *NS1* gene from the viral genome may make the DelNS1 LAIV vaccine a perfect immunogen to prime the host, maximizing the spectrum of B cell and T cell repertoires recognizing most or all viral components, which may be critical for boosting immune responses to conserved epitopes upon subsequent infection with a similar or novel strain. DelNS1 LAIV has the ability to induce high-level protective humoral and T cell responses to influenza viruses. Our data show that cross protective immunity elicited by CA4-DelNS1 (H1N1) is mediated by long-lasting T cell immunity, in particular, by CD8^+^ T cells (Fig. 3; see also Fig. 6). Importantly, the antibodies induced by CA4-DelNS1 (H1N1) are sufficient to protect mice from lethal challenge with 2009 H1N1 virus, even after depletion of both CD4^+^ and CD8^+^ T cells (Fig. 6). Seasonal H3N2 viruses evolve rapidly, with more H3N2 epidemics being recorded than H1N1 or influenza B virus epidemics in the years since H3N2 emerged in 1968 (46–48). The HK4801/2014 virus is distinct from the HK68 H3N2 strain in terms of antigenicity. However, priming with HK4801/2014 rapidly induces immunity in mice to fully protect them against challenge with HK68-MA virus. One strategy to improve the current influenza vaccine regime is to induce immunities to multiple viral targets. Since NS1 is deleted from the viral genome, there is an opportunity to include other antigens in the vaccine strain; we have demonstrated that expressing the HA1 domain of influenza B virus in the CA4-DelNS1 system protects immunized mice from challenge with B/Brisbane/60/2008 virus (Fig. 7). The DelNS1 backbones described in this study could therefore be used as viral vectors to express extra antigens to enhance specific immunity against influenza or to serve as vaccines for other respiratory viruses. Further studies are necessary to more precisely define the associated immune responses, in particular, the adaptive immune response, induced by DelNS1 LAIV versus wild-type virus in mice and other animal models. It remains to be investigated whether other biomarkers may more accurately reflect the protective efficacy of DelNS1 LAIVs, and it will also be important to characterize the immune profile induced by DelNS1 LAIVs in humans. This study tested only monovalent DelNS1 vaccines, and further studies to evaluate multivalent vaccines consisting of combinations of DelNS1 live attenuated viruses are necessary. While cross protective activity was observed using individual DelNS1 H1N1, H3N2, and B LAIVs, it may be still necessary to test combinations of H1N1 and H3N2 LAIVs together with LAIVs representing either of the influenza B virus lineages to optimize cross protection against wild-type viruses.

## MATERIALS AND METHODS

### Cells and viruses.

All cell lines were obtained from ATCC. Human embryonic kidney cells (293T) and lung adenocarcinoma cells (A549) were maintained in Dulbecco’s minimal essential medium (DMEM) supplemented with 10% fetal bovine serum, 100 units/ml penicillin, and 100 μg/ml streptomycin sulfate (P/S) (Life Technologies). Canine MDCK cells were cultured in Eagle’s minimal essential medium (MEM) supplemented with the same amount of serum and antibiotics. H1N1 virus A/California/04/2009, H5N1 virus A/Vietnam/1194/2004, H7N9 virus A/Zhejiang/DTID-ZJU01/2013, and influenza B virus B/HK/8038/2011 were rescued by reverse genetics (49, 50). CA4-DelNS1-HK4801 (H3N2) was made with HA and NA derived from A//Hong Kong/4801/2014 virus (GenBank accession no. EPI1026704-11). CA4-DelNS1 (H1N1), WSN-DelNS1 (H1N1), CA4-DelNS1-HK4801 (H3N2), H7N9-DelNS1, and B-DelNS1 (influenza B) LAIVs were constructed and rescued according to the protocols described here and in our previous report (31). CA4-DelNS1-BHA1 and CA4-DelNS1-HK4801-BHA1 were constructed to express HA1 of B/Brisbane/60/2008 in the NS1 site of either the CA4-DelNS1 (H1N1) or CA4-DelNS1-HK4801 LAIV backbone. Viral gene segments were amplified and cloned into pHW2000 plasmids, resulting in eight pHW2000 plasmids, which were transfected into 293T/MDCK cell mixtures. Rescued virus was amplified in MDCK cells or embryonated chicken eggs. Mouse-adapted H1N1 A/California/04/2009, H3N2 A/HK/1/1968, B/Florida/4/2006, and B/Brisbane/60/2008 viruses were as described previously (35–37, 51). Cold-adapted live attenuated influenza vaccine (ca-LAIV-H1N1) was purified from the Influenza Mist LAIV vaccine available in the 2012 influenza season.

### Construction of plasmids.

NS1 deletion plasmid pHW2000-DelNS1 was constructed as described before (31). Inverse PCR was performed to delete the NS1 gene using plasmid pHW2000-CA4-NS (influenza A virus) or plasmid pHW2000-8038-NS (influenza B virus) as a template. The PCR product was then gel purified, phosphorylated, and self-ligated using a standard protocol. Primers for CA4-DeNS1 inverse PCR were 5′-GACATACTTATGAGGATGTC-3′ (CA4-DelNS1-F) and 5′-CTGAAAGCTTGACATGGTGTTG-3′ (CA4-DelNS1-R). Primers for influenza B DelNS1 inverse PCR were 5′-CTCAATTTGTGTTGTGGTCATGTTGTCCGCC-3′ (Influenza b-DelNS1-F) and 5′-TGGAGGATGAAGAAGATGGCCATCGGATCCTC-3′ (Influenza b-DelNS1-R). A QuikChange II site-directed mutagenesis kit (Agilent) was used to generate point mutations.

### Generation and passage of DelNS1 viruses.

Eight pHW2000 plasmids containing the DelNS1 segment and the other 7 influenza virus genomic segments, together with an NS1 expression plasmid, were transfected into a 293T/MDCK cell mixture and incubated overnight. The DNA mixture was removed and MEM supplemented with 1 μg/ml N-tosyl-l-phenylalanine chloromethyl ketone (TPCK)-treated trypsin (Sigma) added. Virus supernatant was collected 72 h later and designated passage 0 (P0) virus and was subsequently passaged in MDCK cells or embryonated chicken eggs. For CA4-DelNS1 virus, rescued virus was passaged in MDCK cells 10 times at 37°C and then a further 10 times at 30°C. For the H3N2 reassortant DelNS1 viruses of CA4-DelNS1-HK4801 and CA4-DelNS1-HK68, 6 plasmids containing CA4-DelNS1 internal genes and H3N2 HA and NA plasmids derived from either A/Hong Kong/4801/2014 or A/Hong Kong/1/1968 virus were used for virus rescue. For influenza B DelNS1 virus DelNS1-B8038, rescued virus was passaged 10 times at 33°C in MDCK cells. For all DelNS1 viruses, deletion of the NS1 gene was confirmed by reverse transcription-PCR (RT-PCR) and sequencing. After several passages, the genomes of DelNS1 viruses were sequenced to identify adaptive mutations.

### Growth kinetics.

Confluent cells (A549 or MDCK) seeded in 24-well plates were infected with viruses at the indicated multiplicity of infection (MOI). After 1 h of adsorption, the viral supernatant was removed and the cells were washed twice with phosphate-buffered saline (PBS) and then overlaid with MEM containing 1 μg/ml TPCK-treated trypsin and incubated at the indicated temperature. Supernatants were collected at different time points, and titers were determined by plaque assay in TPCK-treated MDCK cells.

### Plaque assay.

Viruses were 10-fold serially diluted and added to confluent MDCK cells in 6-well plates and then incubated at 37°C for 1 h. The supernatant was removed, and the cells were washed twice with PBS and then overlaid with 1% MEM agarose containing 1 μg/ml TPCK-treated trypsin. The plates were incubated at 33°C for 48 h and then fixed with 10% PBS–buffered formaldehyde solution for at least 2 h. Plaques were visualized by staining with 1% crystal violet solution.

### Detection of defective interfering (DI) RNA.

Detection of DI RNA of different segments (PB1, PB2, and PA) was done according to a previously described method (34). For comparisons, passaged CA4-DelNS1, commercial coadapted Flumist live attenuated influenza vaccine (2014–2015 season), plaque-purified cold-adapted H1N1 CA7 from Flumist, and passaged Flu B-DelNS1 were examined. RNA was extracted by using a viral RNA extraction kit (Qiagen). Viral RNAs were subjected to reverse transcription using a High-Capacity cDNA synthesis kit (Invitrogen) and Uni-12 (Flu A) or Uni-9 (Flu B). Segment-specific viral genes were amplified using Hot start *Taq* (TaKaRa) and long-segment-specific primers. The NS segment was also amplified as a control. PCR products were resolved by the use of 1.5% agarose gels and stained using gel red (Biotium). Images were captured by an Azure 150 system (Azure).

### Mouse vaccination and challenge.

Female BALB/c mice (4 to 6 weeks old) were obtained from the Laboratory Animal Unit, the University of Hong Kong. Mice were vaccinated intranasally with DelNS1 viruses (for CA4-DelNS1, 2 × 10^6^ PFU; for CA4-DelNS1-HK4801, 3 × 10^7^ PFU; for DelNS1-B8038, 2 × 10^6^ PFU; for CA4-DelNS1-HK68, 2 × 10^6^ PFU; for ca-LAIV, 2 × 10^6^ PFU; for CA4-DelNS1-BHA1, 1 × 10^6^ PFU; for CA4-DelNS1-HK4801-BHA1, 1 × 10^7^ PFU) 3 weeks prior to virus challenge. Blood was collected for hemagglutination inhibition assay on the day before virus challenge and from the surviving mice at 14 days postinfection. To test for long-lasting immunity, mice were immunized 10 weeks before challenge with different viruses. For virus challenge, vaccinated mice were inoculated intranasally with the indicated viruses (for mouse-adapted H1N1, 10 MLD_50_, 1 × 10^5^ PFU; for H5N1, 100 MLD_50_, 100 PFU; for H7N9, 10 MLD_50_, 2 × 10^5^ PFU; for mouse-adapted influenza B/Florida/4/2006 virus, 10 MLD_50_, 5 × 10^5^ PFU; for mouse-adapted influenza B/Brisbane/60/2008 virus, 10 MLD_50_, 5 × 10^6^ PFU; for mouse-adapted H3N2 A/HK/1/68 virus, 10 MLD_50_). Body weights, disease symptoms, and survival rates were observed for 14 days. Mice with a body weight loss of greater than 25% were euthanized, in accordance with animal ethics guidelines. To evaluate viral replication in mouse lungs, lungs were collected at day 3 and homogenized in 1 ml PBS. Viral titers were determined by plaque assay in MDCK cells. The protocols used in this experiment were approved by the Committee on the Use of Live Animals in Teaching & Research (CULATR-3064-13), the University of Hong Kong. The CULATR follows Hong Kong legislation-recommended and Association for Assessment and Accreditation of Laboratory Animal Care International (AAALAC)-recommended standards/guidelines (http://www.aaalac.org/about/guidelines.cfm).

### Hemagglutination inhibition (HI) and microneutralization (MN) assays.

Sera from immunized mice were treated with receptor-destroying enzyme (RDE; Merck). Control antibodies and sera were then 2-fold serially diluted in 96-well plates. Subsequently, a total of 8 HA units of virus was added into each well and incubated at room temperature for 1 h, and then 50 μl of 0.5% turkey red blood cells was added to the serum/virus mixture followed by incubation for 30 min. The HI titer is defined as the reciprocal of the highest dilution of sera that completely inhibits hemagglutination. For microneutralization assay, RDE-treated sera were mixed with 100 50% tissue culture infective doses (TCID_50_) of virus and were then incubated for 1 h at 37°C. The mixture was added to confluent MDCK cells in MEM supplemented with 1 μg/ml TPCK-treated trypsin in a 96-well plate and incubated at 37°C for 3 days. Presence of virus was confirmed by hemagglutination (HA) assay. The neutralizing titer is defined as the reciprocal of the highest dilution of sera that completely neutralizes infectivity of 100 TCID_50_ of virus in MDCK cells.

### Intracellular cytokine staining (ICS).

Antigen-specific CD8^+^ and CD4^+^ T cells were examined after *ex vivo* peptide stimulation by ICS. After 3 weeks, vaccinated mice were euthanized; spleens were then removed and homogenized using a cell strainer (BD), and splenocytes were resuspended in DMEM (supplemented with 10% fetal bovine serum [FBS] and P/S). Red blood cells were lysed by addition of lysing solution (BD). Cells were counted and resuspended in DMEM. Cultures (100-μl volumes containing 1 × 10^6^ cells) were grown at 37°C for 1 h with 1 μM NP-specific peptide (GenScript) (for CD4^+^-specific NP, RLIQNSITIERMVLS; for CD8^+^-specific NP, TYQRTRALV) or no peptide. Brefeldin A (BFA) was then added to reach a final concentration of 5 μg/ml, and the cells were incubated for 3 h. Cells were then washed with fluorescence-activated cell sorter (FACS) buffer (2% FBS–PBS) and stained with anti-CD8α-PB, anti-CD4-allophycocyanin (APC), and Zombie (BioLegend) for 30 min at 4°C. Cells were washed and fixed with Perm/Wash buffer (BD) and then stained with anti-gamma interferon (IFN-γ)-phycoerythrin (PE), anti-tumor necrosis factor alpha (TNF-α)-fluorescein isothiocyanate (FITC), and anti-interleukin-2 (IL-2)-PE/Cy7 (BioLegend) overnight at 4°C. Cells were then washed with FACS buffer twice and resuspended in FACS buffer. The samples were acquired with a BD FACSAria III cell sorter and the generated data analyzed with Flowjo V9. Frequencies of IFN-γ-positive (IFN-γ^+^) Zombie-negative (Zombie^−^) CD8^+^ and CD4^+^ T cell populations were determined. Backgrounds were determined using no peptide in stimulations, and the results were subtracted from the values reported.

### Depletion of T cells.

T cell depletion was performed according to previously established protocols (36, 52). Briefly, vaccinated mice were injected intraperitoneally (i.p.) with 100 μg of anti-CD8α antibody (clone 2.43) or anti-CD4 antibody (clone GK1.5) or with both anti-CD8 and anti-CD4 or isotype control (IgG2b) antibodies (Bio X Cell) at days −4, −2, 0, and 3. Depletion of T cells was confirmed at day −1 to ensure greater than 98% depletion compared with the results seen with the isotype control. Mice were challenged with H7N9 virus (10 MLD_50_) at day 0 and monitored for 14 days.

## References

[1] Molinari NA, Ortega-Sanchez IR, Messonnier ML, Thompson WW, Wortley PM, Weintraub E, Bridges CB 2007 The annual impact of seasonal influenza in the US: measuring disease burden and costs. Vaccine 25:5086–5096. doi:10.1016/j.vaccine.2007.03.046. 17544181

[2] Saletti G, Gerlach T, Rimmelzwaan GF 2018 Influenza vaccines: ‘tailor-made’ or ‘one fits all’. Curr Opin Immunol 53:102–110. doi:10.1016/j.coi.2018.04.015. 29734023

[3] Nachbagauer R, Palese P 2018 Development of next generation hemagglutinin-based broadly protective influenza virus vaccines. Curr Opin Immunol 53:51–57. doi:10.1016/j.coi.2018.04.001. 29680576

[4] Petsch B, Schnee M, Vogel AB, Lange E, Hoffmann B, Voss D, Schlake T, Thess A, Kallen KJ, Stitz L, Kramps T 2012 Protective efficacy of in vitro synthesized, specific mRNA vaccines against influenza A virus infection. Nat Biotechnol 30:1210–1216. doi:10.1038/nbt.2436. 23159882

[5] Liebowitz D, Lindbloom JD, Brandl JR, Garg SJ, Tucker SN 2015 High titre neutralising antibodies to influenza after oral tablet immunisation: a phase 1, randomised, placebo-controlled trial. Lancet Infect Dis 15:1041–1048. doi:10.1016/S1473-3099(15)00266-2. 26333337

[6] Baz M, Boonnak K, Paskel M, Santos C, Powell T, Townsend A, Subbarao K 2015 Nonreplicating influenza A virus vaccines confer broad protection against lethal challenge. mBio 6:e01487-15. doi:10.1128/mBio.01487-15. 26489862 PMC4620468

[7] Impagliazzo A, Milder F, Kuipers H, Wagner MV, Zhu X, Hoffman RMB, van Meersbergen R, Huizingh J, Wanningen P, Verspuij J, de Man M, Ding Z, Apetri A, Kükrer B, Sneekes-Vriese E, Tomkiewicz D, Laursen NS, Lee PS, Zakrzewska A, Dekking L, Tolboom J, Tettero L, van Meerten S, Yu W, Koudstaal W, Goudsmit J, Ward AB, Meijberg W, Wilson IA, Radošević K 2015 A stable trimeric influenza hemagglutinin stem as a broadly protective immunogen. Science 349:1301–1306. doi:10.1126/science.aac7263. 26303961

[8] Steel J, Lowen AC, Wang TT, Yondola M, Gao Q, Haye K, Garcia-Sastre A, Palese P 2010 Influenza virus vaccine based on the conserved hemagglutinin stalk domain. mBio 1:e00018-10. doi:10.1128/mBio.00018-10. 20689752 PMC2912658

[9] Chen YQ, Wohlbold TJ, Zheng NY, Huang M, Huang Y, Neu KE, Lee J, Wan H, Rojas KT, Kirkpatrick E, Henry C, Palm AE, Stamper CT, Lan LY, Topham DJ, Treanor J, Wrammert J, Ahmed R, Eichelberger MC, Georgiou G, Krammer F, Wilson PC 2018 Influenza infection in humans induces broadly cross-reactive and protective neuraminidase-reactive antibodies. Cell 173:417–429.e410. doi:10.1016/j.cell.2018.03.030. 29625056 PMC5890936

[10] Wohlbold TJ, Podolsky KA, Chromikova V, Kirkpatrick E, Falconieri V, Meade P, Amanat F, Tan J, tenOever BR, Tan GS, Subramaniam S, Palese P, Krammer F 2017 Broadly protective murine monoclonal antibodies against influenza B virus target highly conserved neuraminidase epitopes. Nat Microbiol 2:1415–1424. doi:10.1038/s41564-017-0011-8. 28827718 PMC5819343

[11] El Bakkouri K, Descamps F, De Filette M, Smet A, Festjens E, Birkett A, Van Rooijen N, Verbeek S, Fiers W, Saelens X 2011 Universal vaccine based on ectodomain of matrix protein 2 of influenza A: Fc receptors and alveolar macrophages mediate protection. J Immunol 186:1022–1031. doi:10.4049/jimmunol.0902147. 21169548

[12] Kolpe A, Schepens B, Fiers W, Saelens X 2017 M2-based influenza vaccines: recent advances and clinical potential. Expert Rev Vaccines 16:123–136. doi:10.1080/14760584.2017.1240041. 27653543

[13] Belshe RB, Mendelman PM, Treanor J, King J, Gruber WC, Piedra P, Bernstein DI, Hayden FG, Kotloff K, Zangwill K, Iacuzio D, Wolff M 1998 The efficacy of live attenuated, cold-adapted, trivalent, intranasal influenzavirus vaccine in children. N Engl J Med 338:1405–1412. doi:10.1056/NEJM199805143382002. 9580647

[14] Du Y, Xin L, Shi Y, Zhang TH, Wu NC, Dai L, Gong D, Brar G, Shu S, Luo J, Reiley W, Tseng YW, Bai H, Wu TT, Wang J, Shu Y, Sun R 2018 Genome-wide identification of interferon-sensitive mutations enables influenza vaccine design. Science 359:290–296. doi:10.1126/science.aan8806. 29348231

[15] Si L, Xu H, Zhou X, Zhang Z, Tian Z, Wang Y, Wu Y, Zhang B, Niu Z, Zhang C, Fu G, Xiao S, Xia Q, Zhang L, Zhou D 2016 Generation of influenza A viruses as live but replication-incompetent virus vaccines. Science 354:1170–1173. doi:10.1126/science.aah5869. 27934767

[16] Wang L, Liu SY, Chen HW, Xu J, Chapon M, Zhang T, Zhou F, Wang YE, Quanquin N, Wang G, Tian X, He Z, Liu L, Yu W, Sanchez DJ, Liang Y, Jiang T, Modlin R, Bloom BR, Li Q, Deng JC, Zhou P, Qin FX, Cheng G 2017 Generation of a live attenuated influenza vaccine that elicits broad protection in mice and ferrets. Cell Host Microbe 21:334–343. doi:10.1016/j.chom.2017.02.007. 28279345

[17] Krug RM 2015 Functions of the influenza A virus NS1 protein in antiviral defense. Curr Opin Virol 12:1–6. doi:10.1016/j.coviro.2015.01.007. 25638592 PMC4470714

[18] Ayllon J, Garcia-Sastre A 2015 The NS1 protein: a multitasking virulence factor. Curr Top Microbiol Immunol 386:73–107. doi:10.1007/82_2014_400. 25007846

[19] Fernandez-Sesma A, Marukian S, Ebersole BJ, Kaminski D, Park MS, Yuen T, Sealfon SC, Garcia-Sastre A, Moran TM 2006 Influenza virus evades innate and adaptive immunity via the NS1 protein. J Virol 80:6295–6304. doi:10.1128/JVI.02381-05. 16775317 PMC1488970

[20] Geiss GK, Salvatore M, Tumpey TM, Carter VS, Wang X, Basler CF, Taubenberger JK, Bumgarner RE, Palese P, Katze MG, Garcia-Sastre A 2002 Cellular transcriptional profiling in influenza A virus-infected lung epithelial cells: the role of the nonstructural NS1 protein in the evasion of the host innate defense and its potential contribution to pandemic influenza. Proc Natl Acad Sci U S A 99:10736–10741. doi:10.1073/pnas.112338099. 12149435 PMC125029

[21] Zanin M, Wong SS, Barman S, Kaewborisuth C, Vogel P, Rubrum A, Darnell D, Marinova-Petkova A, Krauss S, Webby RJ, Webster RG 2017 Molecular basis of mammalian transmissibility of avian H1N1 influenza viruses and their pandemic potential. Proc Natl Acad Sci U S A 114:11217–11222. doi:10.1073/pnas.1713974114. 28874549 PMC5651783

[22] Richt JA, Garcia-Sastre A 2009 Attenuated influenza virus vaccines with modified NS1 proteins. Curr Top Microbiol Immunol 333:177–195. doi:10.1007/978-3-540-92165-3_9. 19768406

[23] Mueller SN, Langley WA, Carnero E, Garcia-Sastre A, Ahmed R 2010 Immunization with live attenuated influenza viruses that express altered NS1 proteins results in potent and protective memory CD8+ T-cell responses. J Virol 84:1847–1855. doi:10.1128/JVI.01317-09. 19939929 PMC2812357

[24] Pica N, Langlois RA, Krammer F, Margine I, Palese P 2012 NS1-truncated live attenuated virus vaccine provides robust protection to aged mice from viral challenge. J Virol 86:10293–10301. doi:10.1128/JVI.01131-12. 22787224 PMC3457311

[25] Richt JA, Lekcharoensuk P, Lager KM, Vincent AL, Loiacono CM, Janke BH, Wu WH, Yoon KJ, Webby RJ, Solorzano A, Garcia-Sastre A 2006 Vaccination of pigs against swine influenza viruses by using an NS1-truncated modified live-virus vaccine. J Virol 80:11009–11018. doi:10.1128/JVI.00787-06. 16943300 PMC1642165

[26] Kappes MA, Sandbulte MR, Platt R, Wang C, Lager KM, Henningson JN, Lorusso A, Vincent AL, Loving CL, Roth JA, Kehrli ME Jr. 2012 Vaccination with NS1-truncated H3N2 swine influenza virus primes T cells and confers cross-protection against an H1N1 heterosubtypic challenge in pigs. Vaccine 30:280–288. doi:10.1016/j.vaccine.2011.10.098. 22067263

[27] Vincent AL, Ma W, Lager KM, Richt JA, Janke BH, Sandbulte MR, Gauger PC, Loving CL, Webby RJ, Garcia-Sastre A 2012 Live attenuated influenza vaccine provides superior protection from heterologous infection in pigs with maternal antibodies without inducing vaccine-associated enhanced respiratory disease. J Virol 86:10597–10605. doi:10.1128/JVI.01439-12. 22811541 PMC3457301

[28] Mossler C, Groiss F, Wolzt M, Wolschek M, Seipelt J, Muster T 2013 Phase I/II trial of a replication-deficient trivalent influenza virus vaccine lacking NS1. Vaccine 31:6194–6200. doi:10.1016/j.vaccine.2013.10.061. 24183981

[29] Wacheck V, Egorov A, Groiss F, Pfeiffer A, Fuereder T, Hoeflmayer D, Kundi M, Popow-Kraupp T, Redlberger-Fritz M, Mueller CA, Cinatl J, Michaelis M, Geiler J, Bergmann M, Romanova J, Roethl E, Morokutti A, Wolschek M, Ferko B, Seipelt J, Dick-Gudenus R, Muster T 2010 A novel type of influenza vaccine: safety and immunogenicity of replication-deficient influenza virus created by deletion of the interferon antagonist NS1. J Infect Dis 201:354–362. doi:10.1086/649428. 20039806

[30] Egorov A, Brandt S, Sereinig S, Romanova J, Ferko B, Katinger D, Grassauer A, Alexandrova G, Katinger H, Muster T 1998 Transfectant influenza A viruses with long deletions in the NS1 protein grow efficiently in Vero cells. J Virol 72:6437–6441. 9658085 10.1128/jvi.72.8.6437-6441.1998 PMC109801

[31] Zheng M, Wang P, Song W, Lau SY, Liu S, Huang X, Mok BW, Liu YC, Chen Y, Yuen KY, Chen H 2015 An A14U substitution in the 3’ noncoding region of the M segment of viral RNA supports replication of influenza virus with an NS1 deletion by modulating alternative splicing of M segment mRNAs. J Virol 89:10273–10285. doi:10.1128/JVI.00919-15. 26223635 PMC4580205

[32] Wressnigg N, Voss D, Wolff T, Romanova J, Ruthsatz T, Mayerhofer I, Reiter M, Nakowitsch S, Humer J, Morokutti A, Muster T, Egorov A, Kittel C 2009 Development of a live-attenuated influenza B DeltaNS1 intranasal vaccine candidate. Vaccine 27:2851–2857. doi:10.1016/j.vaccine.2009.02.087. 19366569

[33] van Wielink R, Harmsen MM, Martens DE, Peeters BP, Wijffels RH, Moormann RJ 2012 Mutations in the M-gene segment can substantially increase replication efficiency of NS1 deletion influenza A virus in MDCK cells. J Virol 86:12341–12350. doi:10.1128/JVI.01725-12. 22951840 PMC3486480

[34] Gould PS, Easton AJ, Dimmock NJ 2017 Live attenuated influenza vaccine contains substantial and unexpected amounts of defective viral genomic RNA. Viruses 9:269. doi:10.3390/v9100269. PMC5691621 28934167

[35] Brown EG, Liu H, Kit LC, Baird S, Nesrallah M 2001 Pattern of mutation in the genome of influenza A virus on adaptation to increased virulence in the mouse lung: identification of functional themes. Proc Natl Acad Sci U S A 98:6883–6888. doi:10.1073/pnas.111165798. 11371620 PMC34447

[36] Valkenburg SA, Li OT, Mak PW, Mok CK, Nicholls JM, Guan Y, Waldmann TA, Peiris JS, Perera LP, Poon LL 2014 IL-15 adjuvanted multivalent vaccinia-based universal influenza vaccine requires CD4^+^ T cells for heterosubtypic protection. Proc Natl Acad Sci U S A 111:5676–5681. doi:10.1073/pnas.1403684111. 24706798 PMC3992686

[37] Shen C, Chen J, Li R, Zhang M, Wang G, Stegalkina S, Zhang L, Chen J, Cao J, Bi X, Anderson SF, Alefantis T, Zhang M, Cai X, Yang K, Zheng Q, Fang M, Yu H, Luo W, Zheng Z, Yuan Q, Zhang J, Wai-Kuo Shih J, Kleanthous H, Chen H, Chen Y, Xia N 18 10 2017, posting date A multimechanistic antibody targeting the receptor binding site potently cross-protects against influenza B viruses. Sci Transl Med doi:10.1126/scitranslmed.aam5752. 29046433

[38] Belongia EA, Simpson MD, King JP, Sundaram ME, Kelley NS, Osterholm MT, McLean HQ 2016 Variable influenza vaccine effectiveness by subtype: a systematic review and meta-analysis of test-negative design studies. Lancet Infect Dis 16:942–951. doi:10.1016/S1473-3099(16)00129-8. 27061888

[39] Houser K, Subbarao K 2015 Influenza vaccines: challenges and solutions. Cell Host Microbe 17:295–300. doi:10.1016/j.chom.2015.02.012. 25766291 PMC4362519

[40] Jin H, Subbarao K 2015 Live attenuated influenza vaccine. Curr Top Microbiol Immunol 386:181–204. doi:10.1007/82_2014_410. 25059893

[41] Murphy BR, Coelingh K 2002 Principles underlying the development and use of live attenuated cold-adapted influenza A and B virus vaccines. Viral Immunol 15:295–323. doi:10.1089/08828240260066242. 12081014

[42] Coelingh KL, Luke CJ, Jin H, Talaat KR 2014 Development of live attenuated influenza vaccines against pandemic influenza strains. Expert Rev Vaccines 13:855–871. doi:10.1586/14760584.2014.922417. 24867587

[43] Heaton NS, Sachs D, Chen CJ, Hai R, Palese P 2013 Genome-wide mutagenesis of influenza virus reveals unique plasticity of the hemagglutinin and NS1 proteins. Proc Natl Acad Sci U S A 110:20248–20253. doi:10.1073/pnas.1320524110. 24277853 PMC3864309

[44] Carrillo B, Choi JM, Bornholdt ZA, Sankaran B, Rice AP, Prasad BV 2014 The influenza A virus protein NS1 displays structural polymorphism. J Virol 88:4113–4122. doi:10.1128/JVI.03692-13. 24478439 PMC3993732

[45] Huang X, Zheng M, Wang P, Mok BW, Liu S, Lau SY, Chen P, Liu YC, Liu H, Chen Y, Song W, Yuen KY, Chen H 2017 An NS-segment exonic splicing enhancer regulates influenza A virus replication in mammalian cells. Nat Commun 8:14751. doi:10.1038/ncomms14751. 28323816 PMC5364394

[46] Fonville JM, Wilks SH, James SL, Fox A, Ventresca M, Aban M, Xue L, Jones TC, Le NMH, Pham QT, Tran ND, Wong Y, Mosterin A, Katzelnick LC, Labonte D, Le TT, van der Net G, Skepner E, Russell CA, Kaplan TD, Rimmelzwaan GF, Masurel N, de Jong JC, Palache A, Beyer WEP, Le QM, Nguyen TH, Wertheim HFL, Hurt AC, Osterhaus A, Barr IG, Fouchier RAM, Horby PW, Smith DJ 2014 Antibody landscapes after influenza virus infection or vaccination. Science 346:996–1000. doi:10.1126/science.1256427. 25414313 PMC4246172

[47] Smith DJ, Lapedes AS, de Jong JC, Bestebroer TM, Rimmelzwaan GF, Osterhaus AD, Fouchier RA 2004 Mapping the antigenic and genetic evolution of influenza virus. Science 305:371–376. doi:10.1126/science.1097211. 15218094

[48] Bedford T, Riley S, Barr IG, Broor S, Chadha M, Cox NJ, Daniels RS, Gunasekaran CP, Hurt AC, Kelso A, Klimov A, Lewis NS, Li X, McCauley JW, Odagiri T, Potdar V, Rambaut A, Shu Y, Skepner E, Smith DJ, Suchard MA, Tashiro M, Wang D, Xu X, Lemey P, Russell CA 2015 Global circulation patterns of seasonal influenza viruses vary with antigenic drift. Nature 523:217–220. doi:10.1038/nature14460. 26053121 PMC4499780

[49] Hoffmann E, Neumann G, Kawaoka Y, Hobom G, Webster RG 2000 A DNA transfection system for generation of influenza A virus from eight plasmids. Proc Natl Acad Sci U S A 97:6108–6113. doi:10.1073/pnas.100133697. 10801978 PMC18566

[50] Song W, Wang P, Mok BW, Lau SY, Huang X, Wu WL, Zheng M, Wen X, Yang S, Chen Y, Li L, Yuen KY, Chen H 2014 The K526R substitution in viral protein PB2 enhances the effects of E627K on influenza virus replication. Nat Commun 5:5509. doi:10.1038/ncomms6509. 25409547 PMC4263149

[51] Zheng B, Chan KH, Zhang AJ, Zhou J, Chan CC, Poon VK, Zhang K, Leung VH, Jin DY, Woo PC, Chan JF, To KK, Chen H, Yuen KY 2010 D225G mutation in hemagglutinin of pandemic influenza H1N1 (2009) virus enhances virulence in mice. Exp Biol Med (Maywood) 235:981–988. doi:10.1258/ebm.2010.010071. 20660098

[52] Guo H, Santiago F, Lambert K, Takimoto T, Topham DJ 2011 T cell-mediated protection against lethal 2009 pandemic H1N1 influenza virus infection in a mouse model. J Virol 85:448–455. doi:10.1128/JVI.01812-10. 20980523 PMC3014175

