# Supplementary material for: Generation of DelNS1 Influenza Viruses: a Strategy for Optimizing Live Attenuated Influenza Vaccines
Source: mBio. 2019 Sep 17;10(5):e02180-19. doi: 10.1128/mBio.02180-19 (PMC6751066; doi:10.1128/mBio.02180-19)
Supplement: FIG S1 [file mBio.02180-19-sf001.pdf]

Fig. S1

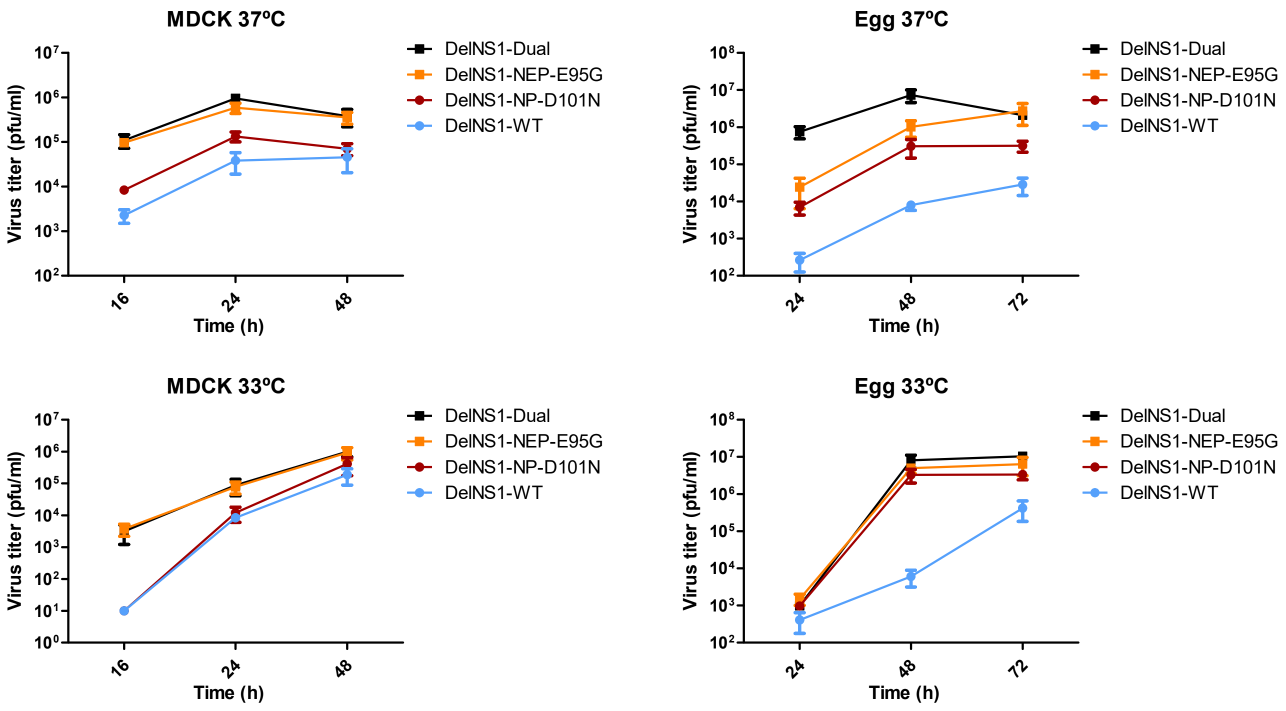

Fig. S1 Effect of the NP-G346A(D101N) and NEP-A310G(E95G) adaptive mutations on the growth of CA4-DelNS1 virus in MDCK cells and embryonated chicken eggs.

MDCK cells or eggs were infected with different versions of CA4-DelNS1 mutants (DelNS1-WT, DelNS1-NP-D101N, DelNS1-NEP-E95G, or DelNS1-Dual) at 0.001 MOI (MDCK) or 100 pfu (eggs). Viral supernatant was collected at the indicated time-points and titrated. Data represents mean values ± standard deviation from three independent experiments.
