# Supplementary material for: Generation of DelNS1 Influenza Viruses: a Strategy for Optimizing Live Attenuated Influenza Vaccines
Source: mBio. 2019 Sep 17;10(5):e02180-19. doi: 10.1128/mBio.02180-19 (PMC6751066; doi:10.1128/mBio.02180-19)
Supplement: FIG S2 [file mBio.02180-19-sf002.pdf]

Fig S2

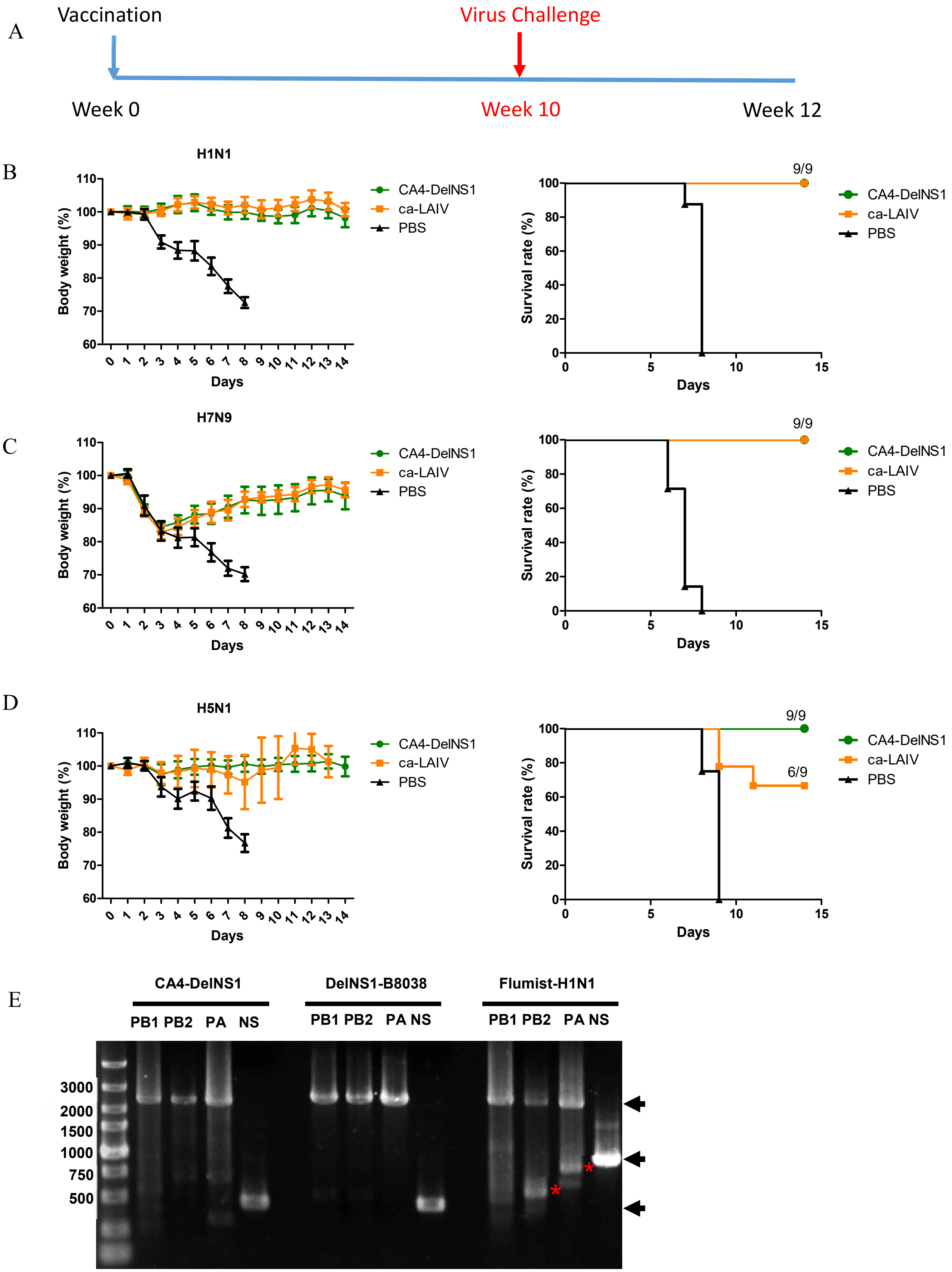
A
Vaccination
Virus Challenge
Week 0
Week 10
Week 12
B
H1N1
Body weight (%)
Days
CA4-DelNS1
ca-LAIV
PBS
Survival rate (%)
9/9
C
H7N9
9/9
D
H5N1
9/9
6/9
E
CA4-DelNS1
DelNS1-B8038
Flumist-H1N1
PB1 PB2 PA NS
3000
2000
1500
1000
750
500

Fig. S2 CA4-DelNS1 LAIV vaccination protects mice from both homologous and heterologous virus challenge.

(A) Timelines for vaccination and virus challenge. Mice were intranasally vaccinated once with either CA4-DelNS1 ($2x10^6$ pfu), ca-LAIV ($2x10^6$ pfu) or PBS then challenged with virus after 10 weeks. Vaccinated mice were challenged with either 10 MLD_{50} CA4 (H1N1) mouse-adapted virus (B), 10 MLD_{50} H7N9 (C), or 100 MLD_{50} H5N1 (D). Body weights and survival were observed for 14 days. Body weight data represents mean values ± standard deviation from 6 mice. (E) Detection of DI RNA in CA4-DelNS1, B-DelNS1, Flumist-H1N1. Gel analysis of the RT-PCR products of segment PB1, PB2, PA and NS of different vaccines. Virus strains were passaged in eggs and viral RNA was extracted and reverse transcribed. Viral polymerase genes and NS segment were amplified by PCR using long segment specific primers. The full length of the polymerase (PB1, PB2 and PA) and NS1 genes was indicated by solid arrowheads and the defective viral genome was indicated by asterisks (*). The NS segment from CA4-DelNS1 and B-DelNS1 viruses contain deletion of NS1. PCR products were resolved in 1.5% agarose gel and stained by gel red and visualized by Azure 150 system.
