# Supplementary material for: Generation of DelNS1 Influenza Viruses: a Strategy for Optimizing Live Attenuated Influenza Vaccines
Source: mBio. 2019 Sep 17;10(5):e02180-19. doi: 10.1128/mBio.02180-19 (PMC6751066; doi:10.1128/mBio.02180-19)
Supplement: FIG S3 [file mBio.02180-19-sf003.pdf]

Fig. S3

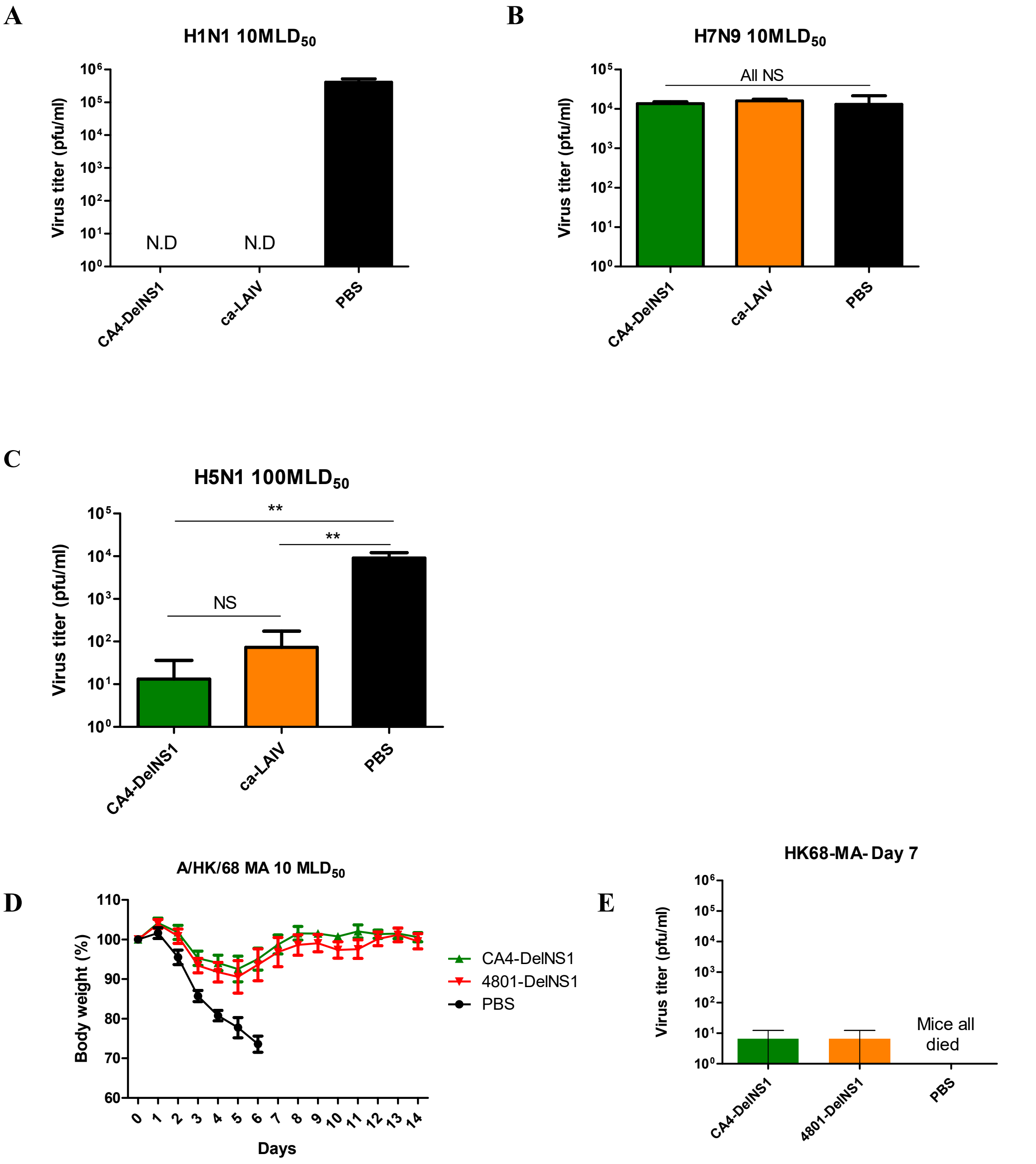

Fig. S3 Viral titers in lungs of mice infected with H1N1, H7N9, H5N1 and H3N2 (HK68) viruses. Mice were vaccinated with CA4-DelNS1 LAIV or ca-LAIV, or mock-immunized with PBS, then three weeks later challenged with mouse-adapted 2009 H1N1 (CA4) (A), or avian H7N9 (B) or H5N1 (C) viruses. Three days after challenge, mice were sacrificed, and lung tissues collected to determine virus titer by plaque assay in MDCK cells. In addition to determining lung virus titers, body weight and survival of groups of 6 mice immunized with CA4-DelNS1 LAIV, CA4-DelNS1-HK4801 or PBS, then challenged with mouse-adapted H3N2 (HK68), were tracked for 14 days (D-E. Lung viral titer data represents mean values ± standard deviation from 3 mice. Statistical analysis between means was performed by Student's t-test: ** $p<0.01$, NS: not significant, N.D: not detected.
