# Supplementary material for: Generation of DelNS1 Influenza Viruses: a Strategy for Optimizing Live Attenuated Influenza Vaccines
Source: mBio. 2019 Sep 17;10(5):e02180-19. doi: 10.1128/mBio.02180-19 (PMC6751066; doi:10.1128/mBio.02180-19)
Supplement: FIG S4 [file mBio.02180-19-sf004.pdf]

Fig. S4

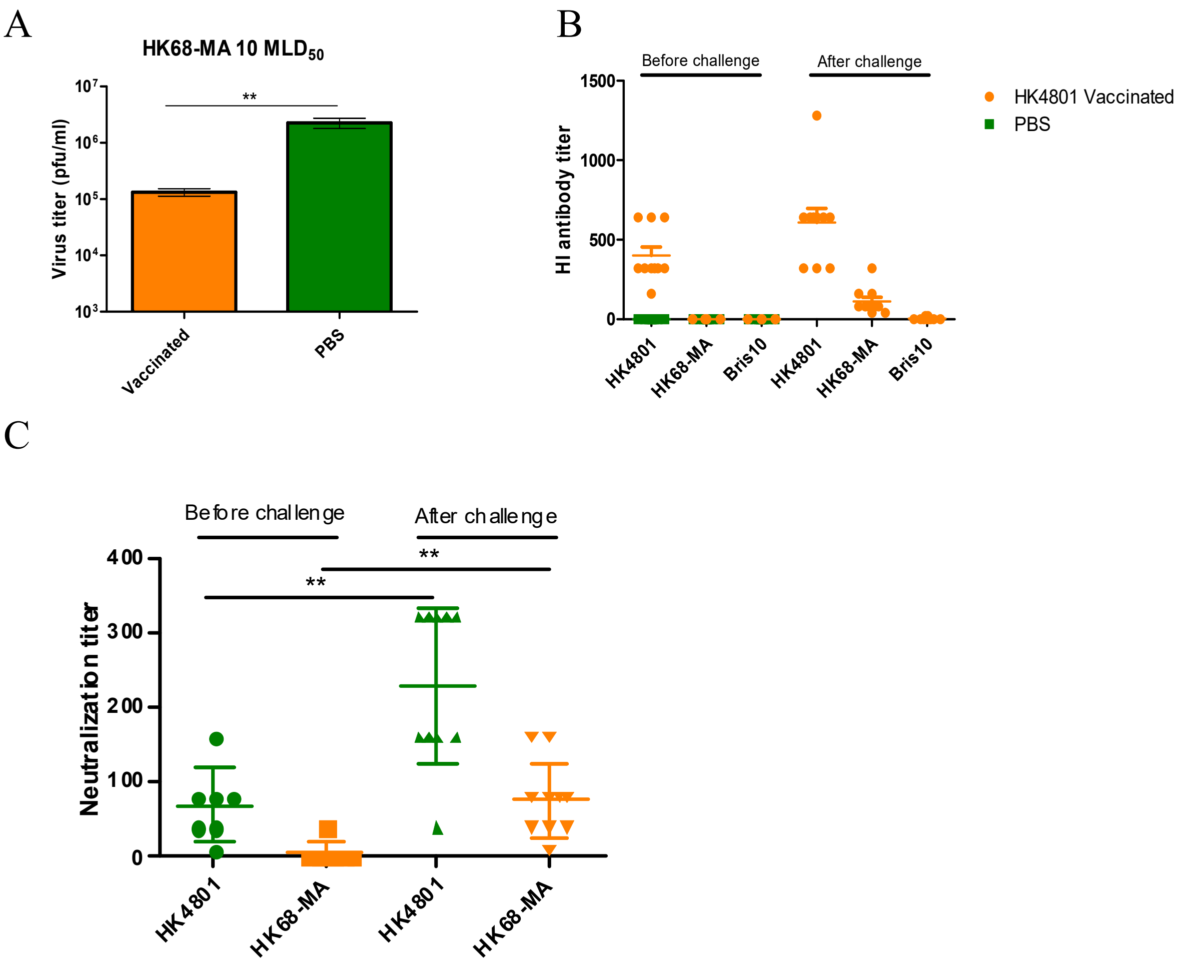

Fig. S4 Viral titers in lungs and antibody response sera from CA4-DelNS1-HK4801 immunized mice challenged with HK68-MA mouse-adapted virus

(A) Mice were vaccinated with CA4-DelNS1-HK4801 (H3N2) or mock immunized with PBS, then after three weeks challenged with HK68-MA mouse-adapted virus. Three days after infection, mice were sacrificed, and lung tissues collected to determine virus titer by plaque assay in MDCK cells. Lung viral titer data represents mean values ± standard deviation from 3 mice. (B) HI analysis of sera from mice vaccinated with CA4-DelNS1-HK4801 or PBS, before and after challenge with HK68-MA. (C) Micro-neutralization assay of sera from mice immunized with CA4-DelNS1-HK4801 and infected with HK68-MA, collected before and on day 14 after challenge. HI and micro-neutralization data represent mean values ± standard deviation from XX mice. Statistical comparisons between means were performed by Student's t-test: ** $p<0.01$.
