# Supplementary material for: Generation of DelNS1 Influenza Viruses: a Strategy for Optimizing Live Attenuated Influenza Vaccines
Source: mBio. 2019 Sep 17;10(5):e02180-19. doi: 10.1128/mBio.02180-19 (PMC6751066; doi:10.1128/mBio.02180-19)
Supplement: FIG S5 [file mBio.02180-19-sf005.pdf]

Fig. S5

A

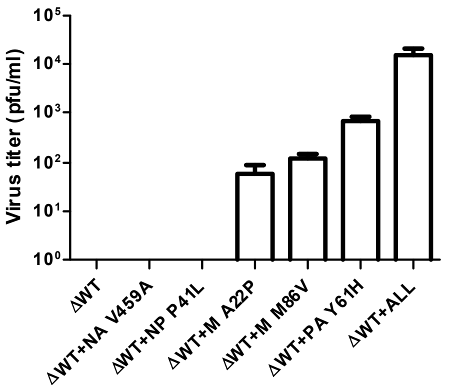

B

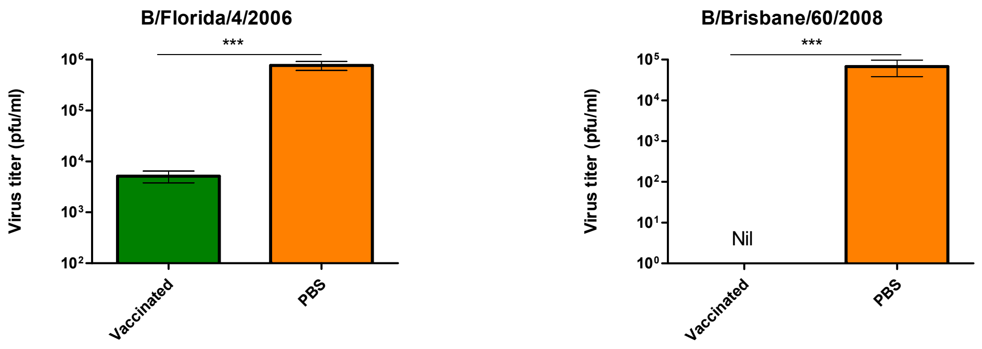

Fig. S5 Viral titers in lungs of mice infected with mouse-adapted influenza B viruses.

(A) Effect of adaptive mutations on efficiency of DelNS1-B8038 virus rescue. DelNS1-B8038 pHW2000 plasmids with individual or combined adaptive mutations or the original DelNS1 plasmid (DelNS1-B8038 WT) were transfected together with plasmids encoding the other 7 viral segments into 293T/MDCK cell mixtures and incubated at 33°C or 37°C. After 72 h, viral supernatants were collected and titrated. Mice were vaccinated with DelNS1-B8038 influenza B LAIV and three weeks later challenged with either Victoria or Yamagata lineage influenza B virus, as indicated in Fig. 5. After 3 days, mice were sacrificed, and lungs collected to determine virus titer by plaque assay. Lung viral titer data represents mean values ± standard deviation from 3 mice. Statistical analysis between means was performed by Student's t-test: *** $p<0.001$.
