# Supplementary material for: Generation of DelNS1 Influenza Viruses: a Strategy for Optimizing Live Attenuated Influenza Vaccines
Source: mBio. 2019 Sep 17;10(5):e02180-19. doi: 10.1128/mBio.02180-19 (PMC6751066; doi:10.1128/mBio.02180-19)
Supplement: FIG S6 [file mBio.02180-19-sf006.pdf]

Fig. S6

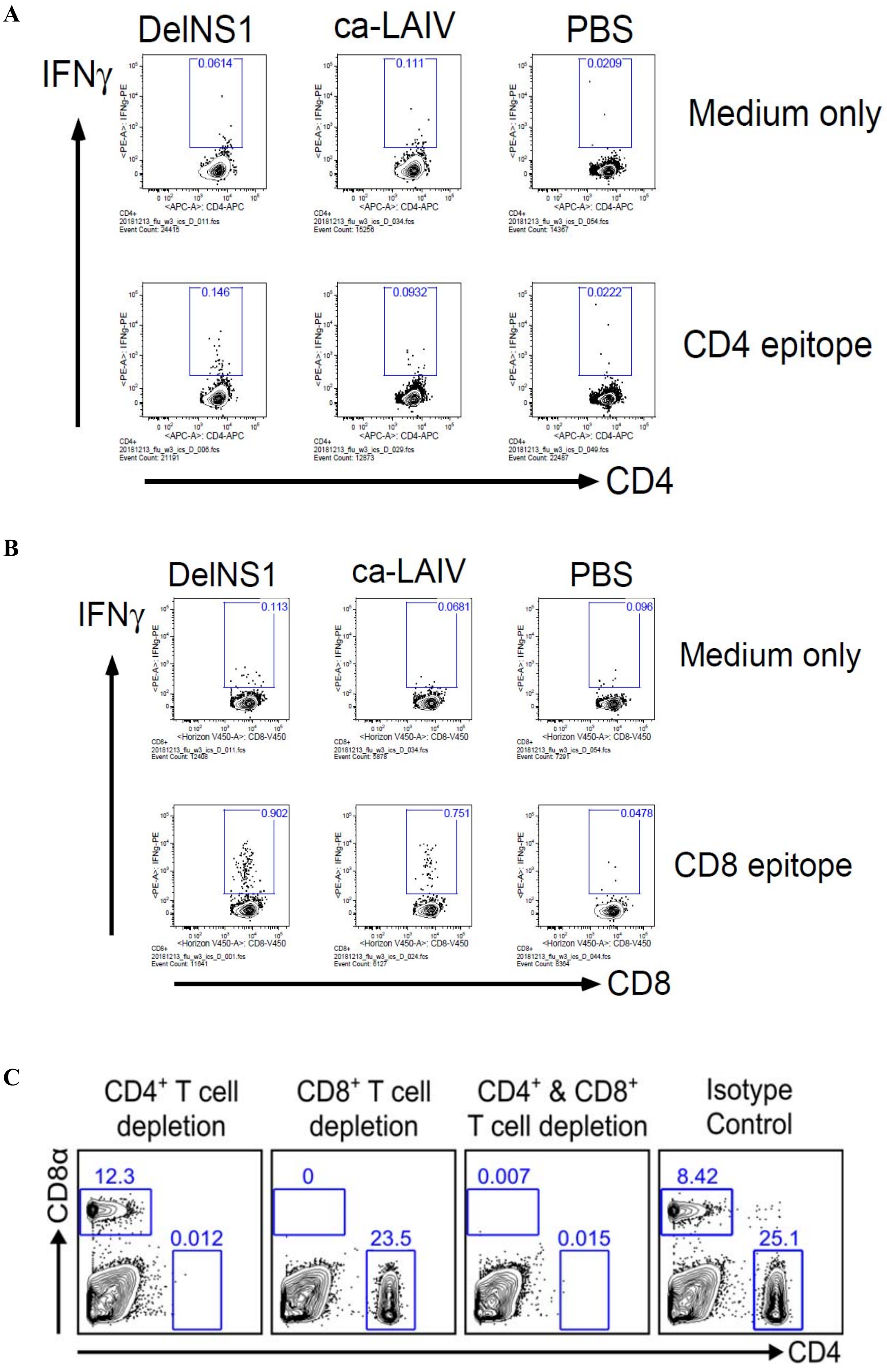
A
DelNS1
ca-LAIV
PBS
IFNγ
Medium only
CD4 epitope
CD4
0.0614
0.111
0.0209
0.146
0.0932
0.0222
B
DelNS1
ca-LAIV
PBS
IFNγ
Medium only
CD8 epitope
CD8
0.113
0.0681
0.096
0.902
0.751
0.0478
C
CD4⁺ T cell depletion
CD8⁺ T cell depletion
CD4⁺ & CD8⁺ T cell depletion
Isotype Control
CD8α
CD4
12.3
0.012
0
23.5
0.007
0.015
8.42
25.1

D

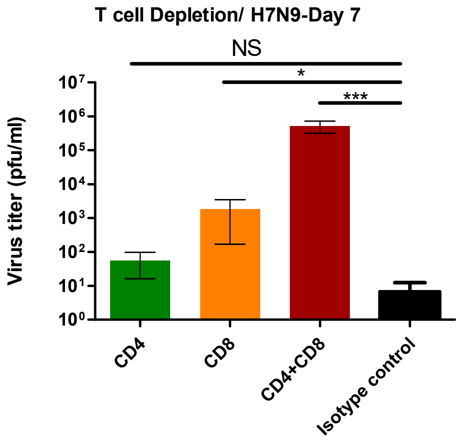

Fig. S6 Depletion of CD4⁺ and CD8⁺ T cells from CA4-DelNS1 LAIV immunized mice and virus titers in lungs of mice challenged with H7N9 virus.

Antigen-specific $CD4^+$ (A) and $CD8^+$ (B) T cell responses were estimated after *ex vivo* peptide stimulation of splenocytes from CA4-DelNS1-, ca-LAIV (H1N1) or mock-vaccinated mice (6 each group) by intracellular cytokine staining (ICS) and flow cytometric analysis. Number inside the box represents frequency of $IFN\gamma^+$ $CD4^+$ or $CD8^+$ T cells. (C) Splenic $CD4^+$ and $CD8^+$ T cell levels in CA4-DelNS1 LAIV vaccinated mice following T cell depletion were measured by intracellular cytokine staining (ICS) and flow cytometric analysis. Vaccinated mice were injected intraperitoneally (i.p) with 100 µg of anti-CD8α, anti-CD4, both anti-CD8α and anti-CD4 or isotype control (IgG2b) antibodies on days 17, 19 and 21 after immunization and day three after virus challenge. Number inside the box represents frequency of $CD4^+$ or $CD8^+$ among total lymphocytes analysed. (D) CA4-DelNS1-vaccinated T cell depleted, and control mice were challenged with H7N9 virus (10 MLD_{50}). After 3 days, mice were sacrificed, and lungs collected to determine virus titer by plaque assay. Percentage of either CD4 or CD8 Lung viral titer data represents mean values ± standard deviation from 3 mice. Statistical analysis between means was performed by Student's t-test: *** $p<0.001$, * $p<0.05$, NS: not significant.
