# Supplementary material for: Generation of DelNS1 Influenza Viruses: a Strategy for Optimizing Live Attenuated Influenza Vaccines
Source: mBio. 2019 Sep 17;10(5):e02180-19. doi: 10.1128/mBio.02180-19 (PMC6751066; doi:10.1128/mBio.02180-19)
Supplement: FIG S7 [file mBio.02180-19-sf007.pdf]

Fig. S7

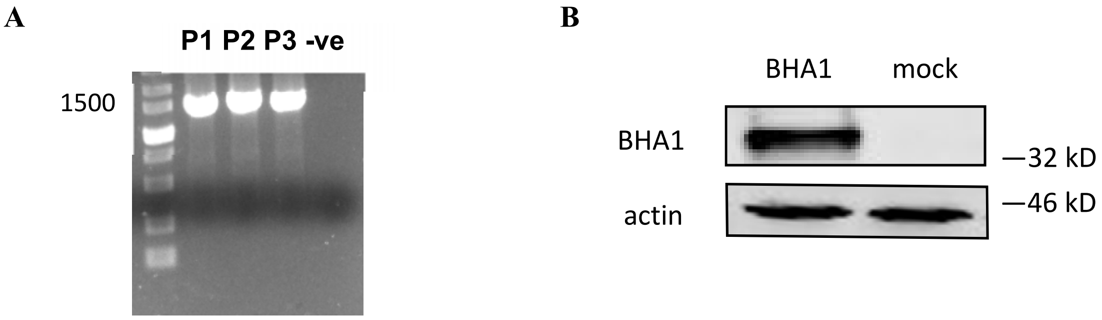

Fig. S7 Construction of CA4-DelNS1 (H1N1) LAIV expressing influenza B HA1

(A) RT-PCR of passages P1-P3 of CA4-DelNS1-BHA1 virus, showing the stability of the BHA1 insert.
(B) Western blot of BHAI expression in lysates of CA4-DelNS1-BHA1 LAIV infected A549 cells.
